# Spin-Electric Effect on a Chiral Dysprosium Complex

**DOI:** 10.1021/jacs.5c10840

**Published:** 2025-08-26

**Authors:** Leonardo Tacconi, Alberto Cini, Arsen Raza, Lorenzo Tesi, Paolo Bartolini, Andrea Taschin, Joris van Slageren, Matteo Briganti, Lorenzo Sorace, Maria Fittipaldi, Mauro Perfetti

**Affiliations:** † Department of Chemistry “Ugo Schiff”, 9300 University of Florence and INSTM Research Unit, Via della Lastruccia 3-13, 50019 Sesto Fiorentino (FI), Italy; ‡ Department of Physics and Astronomy, University of Florence and INSTM Research Unit, Via Sansone 1, 50019 Sesto Fiorentino (FI), Italy; § Department of Industrial Engineering, DIEF, 50139 Florence, Italy; ∥ Institute of Physical Chemistry and Center for Integrated Quantum Science and Technology, 9149 University of Stuttgart, Pfaffenwaldring 55, 70569, Stuttgart, Germany; ⊥ Consiglio Nazionale delle Ricerche, Istituto Nazionale di Ottica, CNR-INO, Via Nello Carrara 1, Sesto Fiorentino (FI), 50019, Italy; # European Lab. for Non-Linear Spectroscopy (LENS), Univ. di Firenze, via N. Carrara 1, I-50019 Sesto Fiorentino (FI), Italy

## Abstract

The Spin-Electric Effect (SEE) on molecules—the influence of external electric fields on molecular spin states—offers a compelling route toward low-power molecule-based spintronic applications. However, SEE remains elusive in molecular systems due to typically weak spin–electric field coupling. In this study, we observe a relevant SEE in a mononuclear lanthanide complex using Electric Field Modulated Electron Paramagnetic Resonance spectroscopy. We reveal a marked anisotropy of the SEE, evidencing that the most perturbed *g* tensor component is the one perpendicular to the electric field, providing hints for the most convenient experimental configuration to tune ad hoc spin transitions. *Ab initio* calculations in synergy with the experimental results revealed that molecular symmetry breaking plays a fundamental role. We also point out the crystal field parameters that are most strongly modulated by the presence of an electric field. These parameters are all off-diagonal, indicating effective electric-field-mediated state mixing.

## Graphical abstract

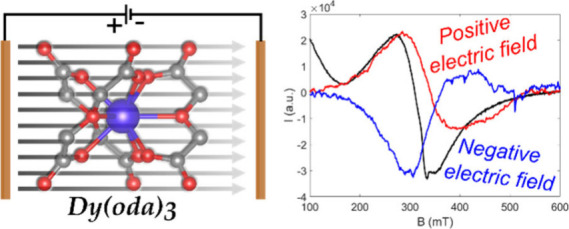

## Introduction

The interaction between magnetic materials and electric fields, known as the magneto-electric effect, holds great promise to create new materials able to sustain the current exponential growth in data production and the increasing demand for high computational power.^1^ The usage of electric fields to control the electron spin provides several technological, strategic, and environmental advantages compared to the current magnetic-field-based technologies. In contrast to magnetic fields, electric fields can be applied on very short length scales; therefore, they have been used to detect,^2^ visualize,^3^ and manipulate^4^ single atoms on the surface. Moreover, they can be simply generated by applying a voltage: a fast, little dissipative and environmentally friendly process compared to the generation of magnetic fields.^5^ The magneto-electric effect has been thoroughly studied in inorganic lattices^6–10^ due to its appealing uses in devices for optical diodes, spin-wave generation, amplification and frequency conversion, modulation of optical waves, and others.^11^

Molecules represent the perfect playground to tailor desired physical properties by modifying chemically adjustable parameters such as bond lengths and angles, elemental constituents, or crystal symmetry. However, in contrast to the widely studied inorganic lattices, the interaction between magnetic molecules and electric fields is poorly studied.^12–18^ The main reason for this gap is that the coupling between molecular spins and electric fields (Spin-Electric Effect, SEE) is generally weak, so that achieving measurable SEE is far from trivial.^10^

Although the exact origin and correct modeling of SEE are still under investigation, recent studies highlighted the importance of molecular properties such as Spin–Orbit Coupling (SOC) and magnetic anisotropy. In this context, Liu et al. suggested that SEEs should scale up linearly with the SOC constant of the paramagnetic ions in the structure.^19^ Indeed, SEEs in lanthanides are usually 1 order of magnitude stronger than in first row transition metals.^20–22^ In addition, SEEs on quasi isotropic metals^23–25^ (*e.g*. Mn^2+^, Fe^3+^) are 1–3 orders of magnitude smaller compared to their anisotropic counterparts.^26^ On the computational side, the most common modeling of SEE presents several challenges related to numerical noise: the molecular systems must be simulated *in silico* under very large electric fields, which are orders of magnitude higher than the experimental ones.^12,27–29^ The proper benchmark of the *ab initio* methodologies requires a significant effort, and the transferability of the approaches to different classes of compounds is not guaranteed. Thus, experimental examples of SEE detected on lanthanide complexes remain rare,^12,30^ and only a few theoretical studies are present in the literature.^12,27,28^ It is also known that magnetically chiral states, encountered for example in frustrated spin triangles with non-negligible antisymmetric interaction, are electric field responsive.^14,18,31^

In this paper, we present the measurement of the SEE detected on single crystals of a trigonal mononuclear chiral lanthanide complex by means of Electric Field Modulated-Electron Paramagnetic Resonance (EFM-EPR) technique.^13^ We show that different SEE values are obtained by applying the electric field along two distinct crystallographic orientations, with the highest one being achieved perpendicularly to the molecular *C*_3_ symmetry axis. By conducting the same experiment on the two enantiomers we also show that topological chirality has no direct impact on the magnitude of the SEE for the studied complexes. A combination of *ab initio* calculations and modeling of the experimental EFM-EPR signal further leads us to conclude that the SEE here observed is among the highest measured to date. Finally, we identify that the main effects of the applied electric field are to induce a higher perturbation in the component of the *g* tensor perpendicular to the applied electric field and to induce distortions of the electronic cloud favoring high mixing of the states.

## Results and Discussion

### Selection of the Investigated Complex

To select the most suitable candidate for our study, we conducted thorough research in the CSD database (version 5.45). Following the literature analysis presented in the Introduction, we looked for a lanthanide-based complex. Molecules containing more than one metal center were excluded, since we aimed to study the SEE arising from a single metal center. Due to our preferred method of investigation (*i.e*. EFM-EPR, see below), the search was further restricted to ions with odd numbers of electrons to maximize the probability of selecting an EPR-active complex.^32^ We targeted structures characterized by collinear magnetic tensors, *i.e*. where the lanthanide ion is located in the highest symmetry point of the lattice. In this condition, all molecules must exhibit the same response to the electric field, simplifying the interpretation of the results. To pinpoint the relation between the SEE and the topological chirality of the molecules, a complex that crystallizes in a chiral space group was required. Among the potential space groups, those exhibiting a high order symmetry axis (*C*_*n*_ with *n* ≥ 3) were favored, as this condition also allows reducing the number of parameters required to describe the Crystal Field (CF) surrounding the metal center.^33^ Finally, the selection was further restricted to easy to synthesize and air stable molecules. In addition to being practical, this requirement has been used to orient the research toward future applications.

The nine-coordinated dysprosium mononuclear complex with 2,2′-oxidiacetic acid (H_2_oda) having the formula Na_3_[Dy­(oda)_3_]­(NaBF_4_)_2_·6H_2_O (CCDC 699737, **Dy­(oda)**_**3**_ hereafter) satisfied all the criteria. The synthesis is achieved through a one-pot reaction, followed by crystallization via slow evaporation of the solvent (water).^34^ The coordination of the lanthanide ion by three ligands occurs in either a right-handed (Δ) or left-handed (Λ) helical fashion. The two enantiomers undergo spontaneous resolution thanks to the cocrystallization salt NaBF_4_, leading to the formation of large hexagonal crystals in the chiral space group *R*32.^34^ The molecular structure is shown, along with the point group symmetry operators in the crystallographic reference frame *abc*, in Figure 1. The *c* and *a* crystallographic axes correspond to the *C*_3_ and *C*_*2*_ symmetry axes of the molecule, respectively. The *R*32 space group is apolar; *i.e*. it is not compatible with the presence of a permanent electric dipole moment.

**Figure 1:**
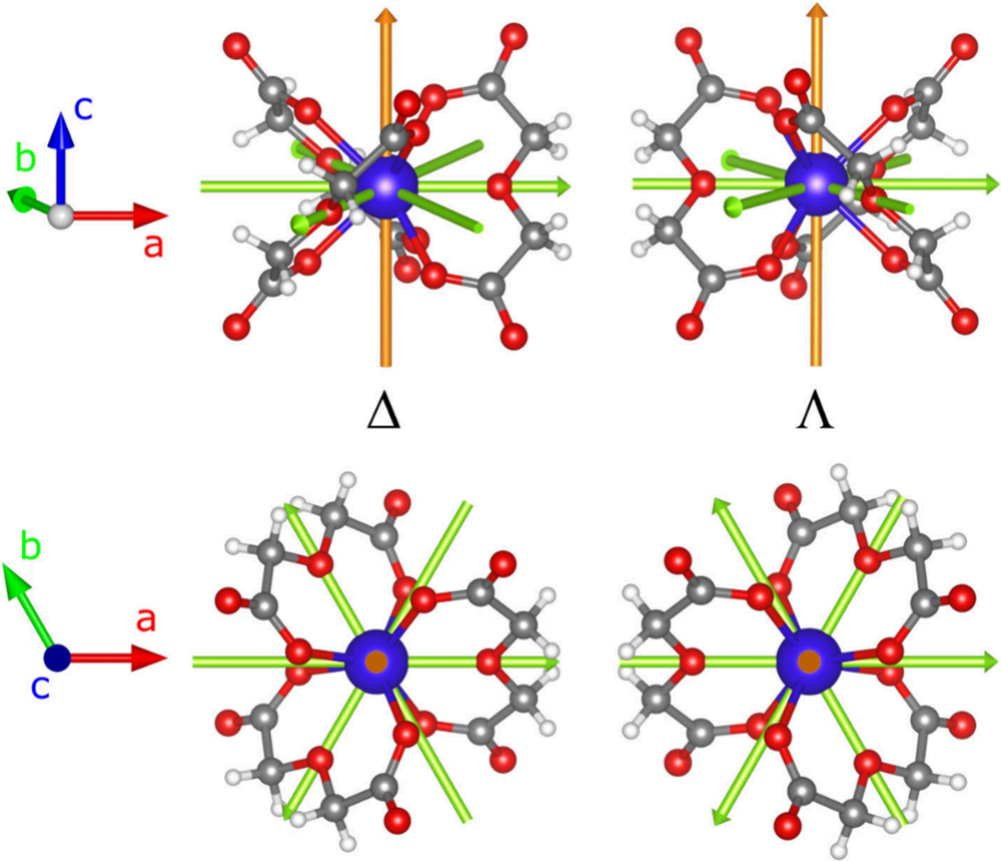
Side and top views of Δ and Λ enantiomers of **Dy­(oda)**_**3**_ with the point group symmetry axis in the *abc* crystallographic reference frame (orange: *C*_*3*_ axis; pale green: *C*_*2*_ axis). Color code: Dy = blue; O = red; C = gray; H = white.

### Magnetometric Characterization

The magnetic properties of the **Dy­(oda)**_**3**_ complex were investigated by using direct current (DC) magnetometry. Prior to magnetic measurements, the purity of the polycrystalline sample was assessed by powder X-ray diffraction (Figure S1a). Temperature-dependent magnetic susceptibility (*χT* vs *T*) measurements were conducted on a polycrystalline sample (see Figure S1b). At room temperature, the *χT* product reaches 14.05 emu K mol^–1^, close to the Curie constant for a Dy^3+^ ion (*J* = 15/2, *g*_*J*_ = 4/3, (*χT*)_*Curie*_ = 14.17 emu K mol^–1^). The monotonic decrease in susceptibility with decreasing temperature can be attributed to the depopulation of energy levels due to CF splitting.^32^ The high symmetry of the complex allowed for magnetometric characterization of oriented single crystals. Consequently, temperature-dependent magnetic susceptibility measurements were also carried out along the two principal crystallographic orientations (*c* and *a* axes) at 100 mT, as shown in Figure 2a. At room temperature, the *χT* values along the *c* and *a* axes are 14.22 and 13.99 emu K mol^–1^, respectively. In the low-temperature regime, the signal along the *c*-axis is higher than the one along the *a*-axis. However, when the temperature is increased, the two curves cross around 12 K. Such behavior, previously observed in lanthanide complexes,^35,36^ is known as magnetic anisotropy switch.^37,38^

**Figure 2:**
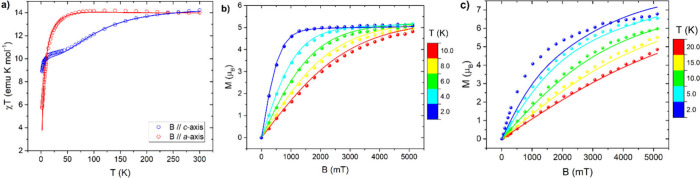
Single crystal DC measurements performed on **Dy­(oda)**_**3**_: *χT* curves acquired at 100 mT along two principal crystallographic orientations *c*-axis (blue) and *a*-axis (red) (a). Temperature dependent magnetization curves acquired along *c*-axis (b) and *a*-axis (c). Dots represent experimental points, and lines represent the simulated curves with the CF parameters obtained from a global fitting procedure, as discussed in the main text.

Temperature and field-dependent magnetization measurements along the two main axes are shown in Figure 2b,c. The low-field, low-temperature signals are consistent with the *χT* measurements, indicating an eas*y*-axis magnetic anisotropy. By increasing the applied magnetic field at low temperatures, the signal becomes higher along the *a*-axis (see Figure S2), meaning that this system also undergoes an in-field magnetic anisotropy switch.^39^ These findings indicate that **Dy­(oda)**_**3**_ exhibits quite a complex and peculiar magnetic anisotropy. In consideration of the easy axis nature of the magnetic anisotropy at low temperatures and low fields, alternating current (AC) susceptibility measurements were also conducted on a polycrystalline sample but no significant slow relaxation could be resolved (see Figure S3). Low-temperature spectroscopic measurements in the terahertz region (see Figure S4) revealed the presence of numerous low-energy vibrational modes, suggesting a phonon-induced fast relaxation.

The magnetic anisotropy of the complex was investigated with Cantilever Torque Magnetometry^40^ (CTM), as shown in Figure S5. The phase change of the signal passing from 5 to 15 K is a direct measurement of the magnetic anisotropy switch, from easy-axis to easy-plane with an increase in temperature, which confirms the single crystal DC magnetometry observations. The high sensitivity of CTM to the orientation of the magnetic reference frame allows extracting fine details about the low temperature behavior, not captured by the DC measurements. While at high temperatures the zero-torque angles correspond to the *c* axis being parallel and perpendicular to the applied field (*i.e*. 0° and 90° in Figure S5), as expected for an axial anisotropy, the low temperature behavior is significantly different. The nonzero torque value when the field is applied in the *ab* plane suggests a spontaneous symmetry breaking. Such behavior will be discussed in more detail in the following section.

### Spectroscopic Characterization

The splitting of the ground *J* = 15/2 Russell–Saunders multiplet of Dy^3+^ was measured using low temperature luminescence spectroscopy, exploiting the ^4^ *F*_9/2_→^6^ *H*_15/2_ transition. Seven peaks out of the expected eight are visible in the spectrum, reported in Figure S6. This observation suggests that at least two doublets are too close to each other to be resolved. The experimentally observed splitting of the ^6^ *H*_15/2_ state is consistent with the results reported by Metcalf and co-workers on the closely related compound Na_3_[Dy­(oda)_3_]_2_·2NaClO_4_·6H_2_O.^41^ This supports the idea that the energy level structure is entirely dependent on the Dy coordination environment.

We initially performed temperature-dependent X-band (ν ≈ 9.4 GHz) EPR experiments on a polycrystalline sample (see Figure S7). Upon decreasing the temperature below 30 K, two main signals can be observed at around 80 and 330 mT, proving the investigated complex to be EPR active. Further decreasing the temperature to 6 K resulted in the emergence of additional features, prompting an investigation into the nature of these peaks. To this end, the experiment was repeated on a single crystal oriented along the two principal crystallographic orientations, the *c* and *a* axes. The resulting spectra at 5 K are presented in Figure 3a. Notably, a single peak is observed along the *c* axis, while two distinct features are discernible along the *a* axis. To ascertain whether the two in-plane signals originate from single center spin transitions or from collective interactions (*e.g.* dipolar), we measured a magnetically diluted sample. First, the Y-based analogue was synthesized to obtain single crystals suitable for X-ray diffraction analysis and structural determination. The resulting crystallographic data and refinement parameters are reported in Table S1. The unit cell dimensions and space group of **Y­(oda)**_**3**_ match those of **Dy­(oda)**_**3**_, confirming the isostructurality. An overlay of the coordination environments around the Dy and Y centers is presented in Figure S8. The average atomic displacement between the two structures is approximately 0.017 Å, highlighting their structural similarity and supporting the feasibility of using **Y­(oda)**_**3**_ as a diamagnetic diluent for **Dy­(oda)**_**3**_. Therefore, a magnetically diluted sample with formula **Y**_**0.99**_ **Dy**_**0.01**_ **(oda)**_**3**_ was synthesized and measured. An angular-dependent experiment, reported in Figure 3b, was conducted on a diluted single crystal from *c* to *a* crystallographic axes at 5 K, which revealed two separate peaks along the *a* axis (*g* = 6.94, *g* = 2.08) collapsing at the same field along the *c* axis (*g* = 9.53). These findings indicate that the observed signals must arise from single spin transitions rather than collective phenomena, hinting toward the presence of a doublet close in energy to the ground state.

**Figure 3:**
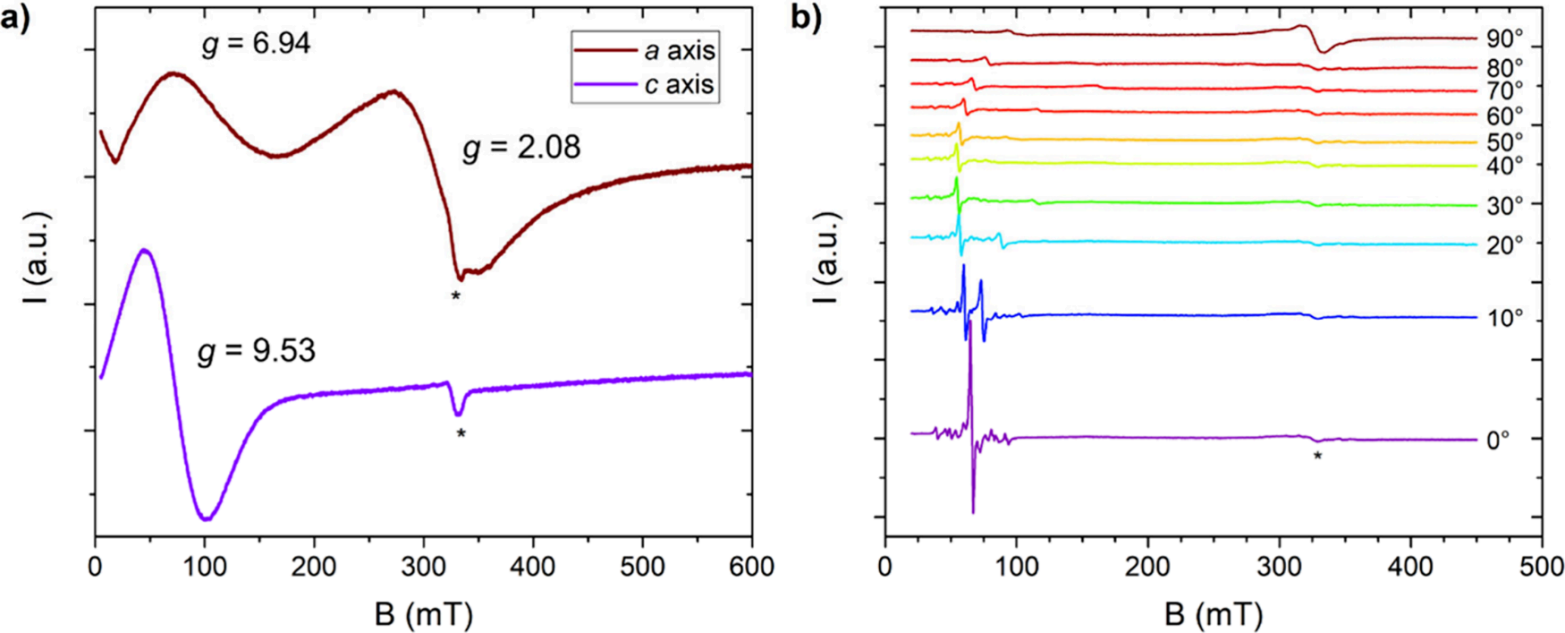
– X-band EPR spectra acquired at 5 K on a single crystal of **Dy­(oda)**_**3**_ along the crystallographic *c*-axis and *a*-axis (a). Angular dependent spectra acquired at 5 K on a single crystal of **Y**_**0.99**_ **Dy**_**0.01**_ **(oda)**_**3**_ from the crystallographic *c*-axis (rotation angle = 0°) to the *a*-axis (rotation angle = 90°) (b). The asterisk indicates the signal arising from the EPR cavity employed for the measurements. The additional features near the low field peaks in (b) can be attributed to hyperfine interactions of the ^161^Dy and ^163^Dy isotopes, both of which have a nuclear spin *I* = 5/2.

### Magnetic Modeling

To obtain a good initial guess to fit the experimental data, *ab initio* calculations were performed. Details about the methodology employed are reported in the Experimental Section. The results of the *ab initio* calculations can be projected on the ground Russell–Saunders multiplet to extract the *B*_*k*_^*q*^ parameters of the corresponding Stevens operators ${Ô}_{k}^{q}\left(J\right)$ .^42^ These can be used in the first term of the following Hamiltonian (eq 1), the CF term. The second term refers to the Zeeman interaction with the applied magnetic field.

1 $${Ĥ}_{4f}=\overset{2,4,6}{\underset{k}{\sum }}\overset{k}{\underset{q=-k}{\sum }}B_{k}^{q}{Ô}_{k}^{q}\left(J\right)+g_{J}\mu_{B}Ĵ\cdot \mathrm{B⃗}$$

The CF parameters obtained from *ab initio* calculations are reported in Table S2, while the resulting energy levels and compositions are shown in Table S3. Figure S6a compares the experimental data with the *ab initio* calculated (see after) CF splitting of the ^6^ *H*_15/2_ multiplet. Notably, *ab initio* calculations underestimate the total splitting, although they predict the presence of a low-lying doublet very close in energy to the ground doublet. By simulating the DC and CTM measurements using these parameters (see Figures S9 and S10, respectively), it is evident that *ab initio* calculations accurately capture the overall electronic structure but they are unable to reproduce all the observed magnetic features. The other two sets of parameters reported in the literature by Baldovì and co-workers^43^ (*ab initio* calculations) and Metcalf and co-workers^41^ (luminescence on Na_3_[Dy­(oda)_3_]_2_·2NaClO_4_·6H_2_O) suffer from the same limitation. This is evident from Figure S11, reporting the simulated *χT* vs *T* curves along the crystallographic *c* axis for the three parameter sets compared to the experimental results. A refined set of CF parameters was therefore sought for by a global fitting procedure of single crystal and powder magnetometric measurements and the energies extracted from luminescence spectroscopy. Only the six CF parameters compatible with the *C*_*3v*_ point group symmetry of the complex were considered: three diagonal (*B*_2_^0^, *B*_4_^0^ and *B*_6_^0^) and three off-diagonal (*B*_4_^3^, *B*_6_^3^ and *B*_6_^6^).^33,44^ The best-fit parameters (Stevens’ notation) are *B*_2_^0^ = −[2.264(76)] × 10^–1^ cm^–1^, *B*_4_^0^ = [6.453(89)] × 10^–3^ cm^–1^, *B*_6_^0^ = −[1.431(73)] × 10^–5^ cm^–1^, *B*_4_^3^ = −[9.650(154)] × 10^–2^ cm^–1^, *B*_6_^3^ = [5.941(109)] × 10^–4^ cm^–1^ and *B*_6_^6^ = −[6.225(162)] × 10^–4^ cm^–1^ (see Table S4 for corresponding values in Wybourne’s notation^42,45^ ).

These provide the eigenfunctions and eigenvalues of **Dy­(oda)**_**3**_ reported in Table S5. Figure S6b shows a comparison between the experimental data and the splitting obtained through a fitting procedure of the experimental data, which demonstrates good agreement with the measurement. The key difference between our energy level structure and the ones obtained by other literature sets is the presence of a low-lying doublet at only 6 cm^–1^. Given that the energy associated with X-band microwave radiation is approximately 0.3 cm^–1^, such a separation of energy precludes X-band EPR allowed interdoublet spin transitions at experimentally accessible fields, and the two experimentally detected transitions must therefore have an intradoublet origin. Furthermore, as shown in Table S5, the calculated Boltzmann population of the two doublets at 5 K is significant, therefore both doublets could give a signal at such a temperature. However, the composition of the ground doublet, dictated by the trigonal nature of the CF, does not satisfy the Δ*m*_*J*_ = ±1 selection rule for EPR spectroscopy, *i.e*. it is expected to be EPR silent. Indeed, the simulation of the angular dependence of the EPR spectrum (Figure S12a) reveals only one observable transition, which is due to the first excited doublet. It is noteworthy that the same conclusions can be drawn by considering the CF parameters obtained from our *ab initio* calculations and those previously reported for **Dy­(oda)**_**3**_ (Tables S3, S6, and S7). A nonzero transition probability in the ground doublet can only be induced by including in the Hamiltonian in eq 1 more parameters than those allowed by strict trigonal symmetry, meaning that the environment surrounding the metal center is slightly distorted. It is noteworthy that the lowering in symmetry upon lowering the temperature in this family of complexes was previously detected in the isostructural Ce-, Er- and Ho-based complexes through EPR^46^ and optical studies.^47,48^ Interestingly, while all these measurements point toward such a scenario, crystal structure investigations have not revealed any kind of phase transition.^49^ Describing such a distortion is not trivial, as, in principle, all 21 out-of-symmetry CF parameters should be considered. As a proof-of-concept that the inclusion of another CF parameter can reproduce the observed EPR signal, we attempted an additional fitting procedure based solely on the angular-dependent EPR spectra acquired from the diluted sample, which included not only the trigonal parameters described previously but also an effective out-of-trigonal symmetry parameter that mimics the distortion. Among the 21 possible parameters, we identified *B*_2_^2^ as the best one to reproduce the trend observed in the experimental angular dependent EPR spectra, as shown in Figure S12b (new set of parameters reported in Table S4). We stress here that the obtained parameter is not necessarily the correct one to include, and that the real distortion is most probably reproduced by the inclusion of several nonzero off-diagonal terms.

### Spin-Electric Characterization

To investigate SEE in a single crystal of **Dy­(oda)**_**3**_, we used EFM-EPR spectroscopy. In a nutshell, EFM-EPR is similar to EPR spectroscopy, except that, in place of the standard magnetic field modulation, an electric field modulation is used (see the Experimental Section for more details). This technique was recently employed by some of us to study the effect of the electric field in several magnetic molecular materials and to extract information on the parameters tuned by the application of the electric field.^13,16,50^ Such a technique is well suited to study oriented single crystals, allowing to address the anisotropy of the SEE.

Initially, the EFM-EPR analysis was conducted on the Δ enantiomer with both the electric and magnetic fields aligned perpendicular to the *C*_3_ axis, pointing parallel to the *b* axis (Figure 4a). This experiment revealed a clear signal, indicating an effective coupling between the electron spin and the applied electric field. By changing the polarity of the electric field, we observed a phase inversion of the signal, confirming that the signal originates from the interaction between the sample and the electric field. The same experiment was then conducted on the Λ isomer (Figure 4b) highlighting that, under identical conditions of electric field polarity, the signal exhibits an opposite phase compared to the Δ enantiomer. Each EFM-EPR spectrum shows a slight asymmetric shape, with a line width broader than that of the standard EPR one, suggesting that the applied electric field influences the relaxation times of the spin system. Moreover, the center of the EFM-EPR signal is shifted by ca. 30 mT compared to the EPR one.

**Figure 4:**
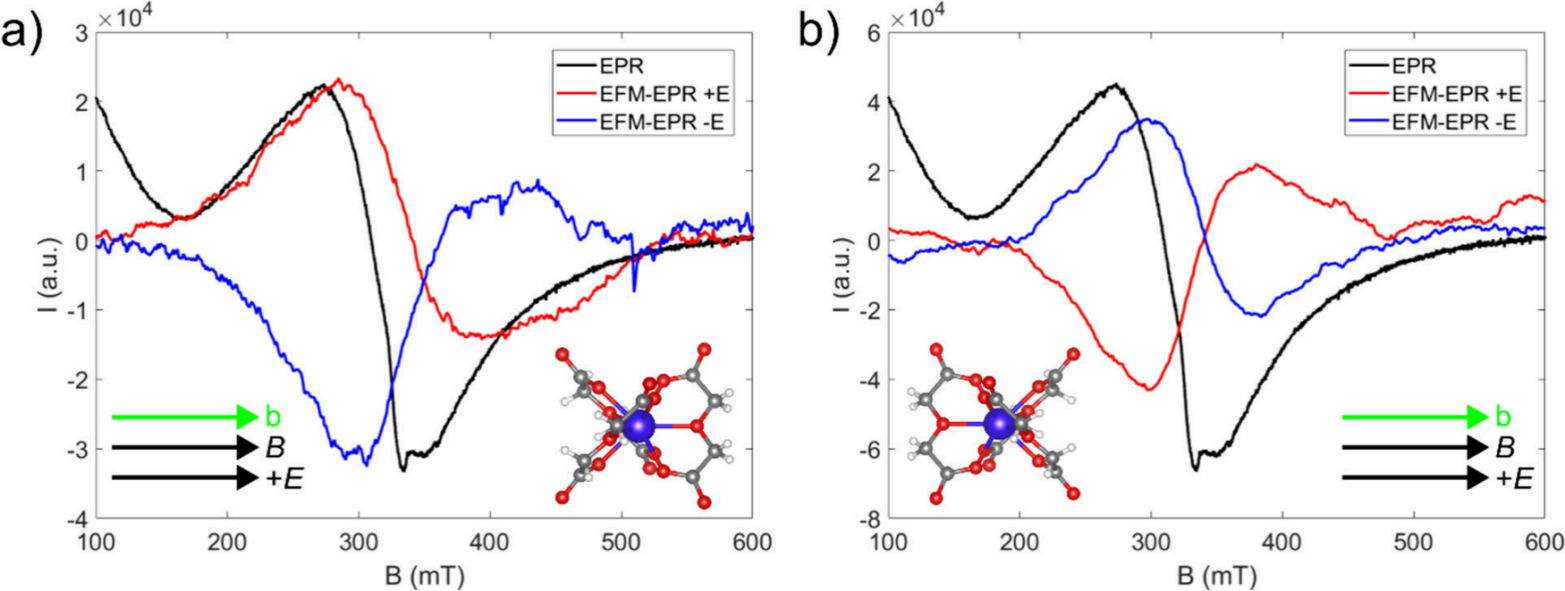
EFM-EPR spectra acquired at 15 K applying the electric (86 kV/m) and magnetic fields parallel to the *b* axis on the Δ (a) and Λ (b) enantiomers. The number of collected acquisitions was: 1 (blue line) and 2 (red line) for Δ enantiomer; 6 (blue line) and 5 (red line) for Λ enantiomer. The corresponding EPR spectra are also shown (black lines), appropriately rescaled to facilitate the comparison with the EFM-EPR spectra.

On the Δ enantiomer, the EFM-EPR analysis was also conducted with the magnetic and electric fields aligned along the *C*_3_ axis, pointing parallel to the *c* axis. A peak undergoing a phase reversal upon inverting the polarity of the applied electric field is observed (Figure S13a), indicating that SEE is also active along this direction. Since the symmetry constraints of the crystallographic *C*_*3v*_ point symmetry would forbid the onset of an EFM-EPR signal in this direction, this observation is another proof that the complex undergoes a spontaneous symmetry breaking at low temperature, as suggested by single crystal EPR and CTM measurements.

Lastly, additional experiments on the Δ enantiomer were performed with electric and magnetic fields being applied perpendicular to each other (Figure S13b). Even in this case, signals that reverse their phases upon changing the polarity of the applied electric field are observed, confirming the occurrence of SEE.

To obtain information on the magnitude of this effect, we undertook simulations of both the standard EPR and the EFM-EPR spectra (see the Experimental Section for more details) considering that the application of an electric field can be associated with a modulation of a specific parameter of the Hamiltonian describing the system. The EFM-EPR simulation is obtained as the difference between the EPR spectra simulated by varying a certain parameter (*i.e.*, positive–negative variation or *vice versa*). In a first attempt to quantify the observed experimental SEE, and compare it with existing literature, we considered that the applied electric field induces a variation of the *g*_*J*_ factor of the Dy^3+^ ion, so that the Hamiltonian in eq 1 can be rewritten as

2 $${Ĥ}_{SEE}=\overset{2,4,6}{\underset{k}{\sum }}\overset{k}{\underset{q=-k}{\sum }}B_{k}^{q}{Ô}_{k}^{q}\left(J\right)+g_{J}\left(E\right)\mu_{B}Ĵ\cdot \mathrm{B⃗}$$

Here, the modulation of the *g*_*J*_ parameter by the applied electric field can be described as follows.

3 $$g_{J}\left(E\right)=g_{J}+\Delta g_{J}\left(E\right)$$

Therefore, the experimental spectra were simulated considering a Δ*g*_*J*_(*E*) able to reproduce the intensity (*i.e.*, the difference between maximum and minimum) of the observed EFM-EPR signal with a proper line width. The parameters necessary to reproduce the experimental spectra are listed in Table 1, while the simulations are shown in Figure S14. In the limit of this model, taking into account an unequal signal-to-noise ratio of the EFM-EPR spectra for the different experimental orientations, Δ*g*_*J*_/*E* results to be strongest when the electric field is applied along *b*, while the effect decreases by 1 order of magnitude when it is applied along *c*. Notably, the application of the electric field along the *b* axis, *i.e.* perpendicularly to the *C*_3_ symmetry axis, leads to one of the largest effects observed to date (see Table 2 for a comparison with Δ*g*_*J*_/*E* values reported in the literature for other molecular complexes). This finding is in agreement with a recent publication by Morrillo *et al*.,^28^ where a large coupling with the electric field was simulated in the direction perpendicular to the pseudosymmetry axis of a mononuclear lanthanide complex. Furthermore, the order of magnitude of the detected effect is consistent with that obtained from another lanthanide-based material^12^ and exceeds that obtained in transition metal-based materials by 1 order of magnitude. Interestingly, when applying the electric field along the *C*_3_ symmetry axis, *i.e.* the *c* axis, the SEE becomes comparable to that typically observed in transition metal complexes.

**Table 1:** Parameters Used to Simulate EPR and EFM-EPR Spectra Acquired on Single Crystal of **Dy­(oda)**_**3**_ with Different Combinations of Orientations of Applied Electric and Magnetic Fields, Considering a Modulation of the *g*_*J*_ Factor^a^

Enantiomer	*B* orientation	*E* orientation	Γ_*EPR*_ (mT)	Γ_*EFM‑EPR*_ (mT)	Δ*g*_*J*_/*E* (10^–9^ m/V)
Λ	∥ *b*	∥ *b*	72.0	83.0	–2.0 ± 0.2
Δ	∥ *b*	∥ *b*	72.0	93.2	1.7 ± 0.2
Δ	∥ *c*	∥ *c*	49.0	70.0	0.7 ± 0.1
Δ	∥ *c*	∥ *b*	49.0	66.4	4.6 ± 0.2

a Γ is the line width peak-to-peak. The error on Δ*g*_*E*_/*E* was evaluated considering a variation of the line width of ±100 G.

**Table 2:** SEE Quantified as Δ*g*/*E* for Other Complexes^a^

Compound	Δ*g*/*E* (10^–9^ m/V)
[Cu_3_(saltag)(py)_6_]ClO_4_ ^15^	0.36
[Co_3_(pytag)(py)_6_Cl_3_]ClO_4_ ^16^	0.11
MnPhOMe^13^ ^,^ ^b^	0.17
[Cu_3_(saltag)(py)_6_]ClO_4_ ^50^ ^,^ ^b^	0.10
[Fe_3_O(PhCOO)_6_(py)_3_]ClO_4_ ^14^ ^,^ ^d^	0.59
Na_9_[Ho(W_5_O_18_)_2_][Bibr ref12] ^,^ ^c^ ^,^ ^d^	1.4

a Crystallization solvents have been omitted for clarity. For transition metal complexes, the value of *g* was considered.

b In this compound the origin of the SEE involves a modulation of the isotropic coupling constant.

c Δ*g*/*E* for Na_9_[Ho­(W_5_O_18_)_2_]·35H_2_O was evaluated from the relation $\frac{\Delta g}{E}=\frac{\Delta \nu }{\nu }\cdot \frac{g}{E}$ and considering *g* = *g*_*J*_ = 5/4, *ν* = 9.40 GHz and Δ*ν* = 11.4 Hz V^–1^ m^12^.

d The effect in these works was evaluated as a change in resonance frequency.

Although a modulation of the *g*_*J*_ factor is a simplification for comparing the SEE with previous reports neglecting their specific origin, it is not ideal for describing such an effect in rare-earth ions. Indeed, *g*_*J*_ is derived by the Landè formula that assumes strong SOC, which is characteristic of free rare-earth ions, and therefore remains largely unaltered by the local CF.^42^ Consequently, its modulation does not adequately capture the influence of the electric field on the CF of the system.

A more accurate description that accounts for the anisotropy of the effect can be achieved by adopting an effective *S*_eff_ = 1/2 Hamiltonian and an anisotropic ***g***-tensor. We extracted and used the effective *g* values of the ground and first excited state from the EPR spectrum (see Figure 3). The electric field modulates the anisotropic ***g***-tensor as follows:

4 $${Ĥ}_{SEE}=\mu_{B}Ŝ\cdot g\left(\mathrm{E⃗}\right)\cdot \mathrm{B⃗}$$

5 $$g_{\alpha }\left(E\right)=g_{\alpha }+\Delta g_{\alpha }\left(E\right)$$

where α = *x*, *y, z* and E⃗ is the experimental electric field. It is worth noting that eqs 4 and 5 consider the tensorial character of ***g***, *i.e.* an applied electric field can influence all of its components. Symmetry imposes that the tensor reference frame has the *z* axis along the *c* axis. The position of the *x* and *y* axes is formally irrelevant; however we have fixed the *x* axis to be collinear with the *b* axis to simplify the following treatment. If the modulation of the ***g*** components is sufficiently small compared to the intrinsic line width of the absorption, then along the static magnetic field it may result in a first derivative-shaped EPR signal. Modulations of a ***g*** component along the microwave magnetic field (*B*_1_) modifies the transition probability and therefore produces an absorption-like EFM-EPR signal.^50^ The optimal perturbation of the ***g*** tensor components for each investigated case was determined considering three different key features of the signal: its phase, its intensity, and its asymmetry (*i.e.*, the relative size of the two lobes).

Initially, we considered the case with both the electric and magnetic fields applied along the *b* crystallographic axis for both enantiomers (***E*** ∥ ***B*** ∥*b*). As discussed previously, the transition observed at around 330 mT along this crystallographic orientation, shown in Figure 4, can be attributed to the first excited state. A modulation on Δ*g*_*x*_/*E* = −2.0 × 10^–9^ m/V can effectively reproduce the intensity of the signal detected on the Λ enantiomer but fails in reproducing its asymmetry (Figure S15a). Conversely, including both modulations of Δ*g*_*x*_/*E* = −1.9 × 10^–9^ m/V and Δ*g*_*z*_/*E* = −15 × 10^–9^ m/V can reproduce the observed signal (Figure 5a). The magnitude of the Δ*g*_*x*_/*E* modulation agrees well with the *ab initio* results even if a much bigger value of Δ*g*_*z*_/*E* is found (see below). Similar results are obtained for the Δ enantiomer. In this case, the intensity of the signal can be reproduced by considering a modulation on Δ*g*_*x*_/*E* = 1.6 × 10^–9^ m/V (see Figure S15b) but to reproduce both the intensity and asymmetry the combination of Δ*g*_*x*_/*E* = 1.4 × 10^–9^ m/V and Δ*g*_*z*_/*E* = 10 × 10^–9^ m/V must be considered (Figure 5b).

**Figure 5:**
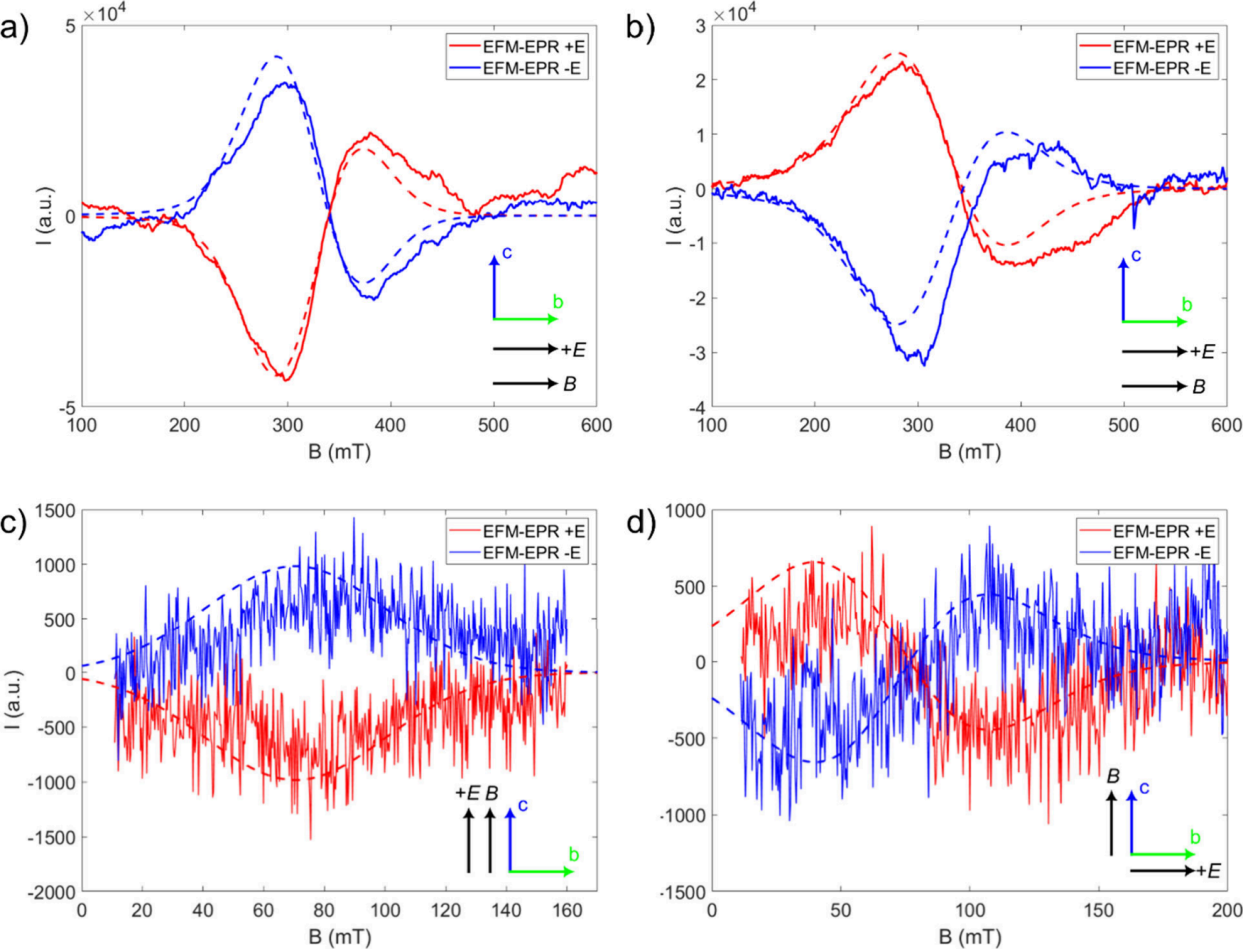
Experimental (solid lines) and simulated (dashed lines) EFM-EPR spectra considering various combination of orientations for the applied electric and magnetic fields: both parallel to the *b* axis on the Λ enantiomer (a); both parallel to the *b* axis on the Δ enantiomer (b); both parallel to the *c* axis on the Δ enantiomer (c); *B* parallel to the *c* axis and *E* parallel to the *b* axis on the Δ enantiomer (d). Relative orientations of *B* and *E* with respect to the crystallographic reference frame are also depicted in the insets. Simulations were performed considering a modulation of an anisotropic ***g*** factor of the first excited state by the applied electric field, as discussed in text.

The simulation of the SEE when the magnetic field is applied along the crystallographic *c* axis (***E*** ∥ ***B*** ∥*c*) is complicated by the fact that both the ground and first excited doublets are EPR active along this orientation, with virtually identical *g*_*z*_value. Moreover, in principle, four different parameters, two for each active doublet, could be perturbed making their determination not unique. For this reason, we evaluated the maximum perturbation on the ***g*** factor of each doublet by considering them separately. As a result, the peak shape of the signal can easily be reproduced by assuming Δ*g*_*x*_/*E* to be −1.2 × 10^–10^ and −4 × 10^–11^ m/V for the first excited (Figure 5c) and ground (Figure S16) state, respectively. Adding a modulation of Δ*g*_*z*_ does not lead to a significant improvement of the simulations, therefore we can only estimate an upper limit of Δ*g*_*z*_/*E* to be −1 × 10^–10^ m/V and −3 × 10^–11^ m/V for the first excited and ground state, respectively. Above this value, the simulation significantly differs from the experiment.

On the contrary, the perturbation on the two doublets can be distinguished when the electric field is applied along the *b*-axis (***E*** ∥*b* and ***B*** ∥c), as the modulation induced by the electric field is expected to be independent of the applied magnetic field. In other words, the perturbation on the ***g*** factor of the first excited state can be fixed to that determined by the ***E*** ∥ ***B*** ∥*b* measurement and thus only the modulation of the ***g*** factor of the ground state needs to be determined. The best obtained values are Δ*g*_*x*_/*E* = 3.8 × 10^–9^ m/V and Δ*g*_*z*_/*E* = 7.6 × 10^–9^ m/V, with the corresponding simulation shown in Figure 5d. Table 3 summarizes the obtained values considering the first excited state doublet and highlights a clear trend: the most perturbed *g* value is always perpendicular to the applied electric field.

**Table 3:** Parameters Used to Simulate EPR and EFM-EPR Spectra Acquired on Single Crystal of **Dy­(oda)**_**3**_ with Different Combinations of Orientations of Applied Electric and Magnetic Fields, Using an Effective *S* = 1/2 Hamiltonian Modeling the First Excited State Doublet, as Discussed in the Text^a^

Enantiomer	Doublet	***B*** orientation	***E*** orientation	*Δg*_*X*_/*E* (10^–9^ m/V)	*Δg*_*Z*_/*E* (10^–9^ m/V)
Λ	FED	∥ *b*	∥ *b*	–1.9 ± 0.3	–15 ± 3
Δ	FED	∥ *b*	∥ *b*	1.4 ± 0.3	10 ± 1
Δ	FED	∥ *c*	∥ *c*	–0.12 ± 0.02	*<*0.1
	GD			–0.04 ± 0.02	*<0.03*
Δ	FED	∥ *c*	∥ *b*	1.4^b^	10^b^
	GD			3.8 ± 0.3	7.6 ± 0.8

a The error on Δ*g*_*α*_/*E* was evaluated considering a variation of the line width of ± 10 mT. GD = ground doublet, FED = first excited doublet.

b Parameter fixed to those previously determined from *E*∥*B*∥*b*.

A more complete approach to simulate the SEE in lanthanide-based systems should consider the electrically induced modulation of the CF surrounding the metal ion, which means that the corresponding parameters of Hamiltonian ([Disp-formula eq2]) should be expressed as

6 $$B_{k}^{q}\left(\mathrm{E⃗}\right)=B_{k}^{q}+\Delta B_{k}^{q}\left(\mathrm{E⃗}\right)$$

Nonetheless, the CF in *C1* symmetry is described by 27 different parameters, and getting a glimpse of how the applied electric field can affect each of them can be a demanding endeavor.

To get a preliminary picture of this effect, we therefore decided to employ *ab initio* calculations. The application of an electric field can modify the CF surrounding lanthanide ions owing to two main effects. The first one is the induction of a geometrical distortion of the metal complex, expected to be most relevant in molecules possessing a permanent electric dipole moment, which is not the case for **Dy­(oda)**_**3**_. The second effect is based on the possibility to induce modification in the electronic cloud^29^ surrounding the metal center, which also leads to a change in the CF. From a computational perspective, the first approach yields substantial results only when considering electric fields several orders of magnitude larger than those experimentally applied.^27^ Consequently, we opted to undertake *ab initio* calculations to elucidate the impact of an electric field on the electronic cloud of the metal center by applying *in silico* an electric field of the same order of magnitude of the one applied experimentally. The calculations were performed by applying a positive electric field oppositely to the crystallographic *b*-axis on the Λ enantiomer to evaluate the impact of the SEE on the lanthanide electronic structure. It should be noted that this effect cannot take place when the electric field is parallel to *c*, since the *C*_3_ symmetry would be preserved. The obtained results are reported in Figure S17 and evidence that a linear behavior of the CF parameters with respect to the applied electric field is found only for some of the parameters which are not allowed in trigonal symmetry, whereas for all the others no clear correlation is observed (see Table S8). Particularly pronounced is the change in the *B*_2_^1^ parameter, around 3 × 10^–8^ cm^–1^ m/kV. However, unambiguous linear trends are present in the out-of-symmetry fourth- and sixth-order parameter. Despite the magnitude of the variations being below the convergence threshold, these trends were confirmed by additional calculations with tighter SCF convergence criteria (see Experimental Section). The variation of the ground doublet Δ*g*_α_/*E* associated with such modulations of the CF parameters is also shown in Figure S18. We attempted the simulation of the EFM-EPR spectra acquired applying the magnetic and electric fields along the crystallographic *b*-axis from the linear fit of the *ab initio* CF parameter variations, considering an electric field of 86 kV/m (Figure S19). A factor of 2 on the resultant simulation is needed to match the experimental intensity of the signal, while the phase of the signal and its position are not reproduced. The same factor is needed if only the out-of-symmetry parameters are considered (see Figure S20). We also attempted to evaluate the effect of geometrical distortion when applying the electric field along −*b*. However, no linear trend in the CF parameters was observed (see Figure S21). This is probably due to the large electric field that was necessary to apply to simulate some changes in the geometrical parameters, *i.e.* four orders of magnitude larger than the experimental one. Despite some discrepancies, the effect on the electronic cloud alone allowed for the recovery of the magnitude of SEE, capturing at the same time its physical mechanism.

The *ab initio* results highlight that the precise determination of the perturbation of the CF parameters induced by the electric field is challenging, as it is the low temperature symmetry breaking in zero electric field. For this reason, we attempted to ascertain the most pertinent parameters responsible for the observed experimental signal by exploiting the peculiar features of the EFM-EPR signal. In our previous analysis based on an effective doublet, the Δ*g*_*x*_ parameter stands out as the most relevant in defining the intensity of the simulated EFM-EPR spectra. In principle, a Δ*g*_*x*_ of a specific magnitude can be achieved by adjusting the variation of any CF parameter, as shown in Figure S22. To restrict our choice, we introduced another constraint in our analysis exploiting the asymmetry of the lobes of the experimental EFM-EPR spectra (*i.e.*, the relative size of the lobes of the spectrum). The EFM-EPR spectrum arises from the difference between two absorption spectra for which the applied positive and negative modulated electric field induces a variation in the Spin Hamiltonian parameters with respect to the unperturbed ones (see the Experimental Section). Such a difference may impact not only the resonance field but also the transition probability, and thus the intensity for the two lobes can be different. The observed experimental asymmetry, characterized by the predominance of the left lobe over the right, can be attributed to the subtraction of two absorption spectra having different intensities (see Figure S23), and can be therefore reconducted to a shift concomitant with a variation of the transition probability Δ*P*, defined in eq 7.

7 $$P\left(\Delta g_{x}\right)=P_{0}+\Delta P\left(\Delta g_{x}\right)$$

To reproduce the experimental spectrum, Δ*P*(Δ*g*_*x*_) must be a positive value. Therefore, calculations were performed to determine the optimal value of each Δ*B*_*k*_^*q*^ parameter, with the aim of achieving a Δ*g*_*x*_/*E* value of −1.9 × 10^–9^ m/V (see Table 3), and the associated Δ*P* value. The results for all 27 CF parameters are displayed in Figure S24. Among the 27 parameters, only Δ*B*_4_^4^ = −2.5 × 10^–6^ cm^–1^ results in a positive Δ*P* value and is thus capable of reproducing the experimental lobes asymmetry. Using the optimal Δ*B*_4_^4^ value to simulate the EFM-EPR spectra, the asymmetry of the lobes is correctly reproduced, as can be evidenced by comparing the two lobes of the simulated spectra in Figure S25a. Nonetheless, a significant discrepancy between intensities of experimental and simulated spectra is still present. Such a mismatch can be mitigated by increasing Δ*B*_4_^4^ to −4.3 × 10^–6^ cm^–1^, which leads to a Δ*g*_*x*_/*E* value of −3.1 × 10^–9^ m/V and the results shown in Figure S25b. This discrepancy must be attributed to the approximation that is intrinsic to the effective spin model. Indeed, a key factor like the transition probabilities between the levels, due to the real compositions of the levels, is not considered.

Although the introduction of Δ*B*_4_^4^ allows reproducing the spectra asymmetry, it is still not sufficient to replicate the position of the signal in the EFM-EPR spectra. The reason is that the modulation of Δ*B*_4_^4^ does not significantly change the *g*_*z*_component (Δ*g*_*z*_/*E* = −4.9 × 10^–12^ m/V, *i.e.*, 3 orders of magnitude lower than the variation estimated using the *S*_eff_ = 1/2 model). Therefore, we then calculated the variation of both Δ*g*_*x*_/*E* and Δ*g*_*z*_/*E* as a function of the simultaneous perturbation of *B*_4_^4^ and each of the others *B*_*k*_^*q*^ parameters, starting from the unperturbed CF Hamiltonian. Results of the calculated modulations of Δ*g*_*x*_/*E* and Δ*g*_*z*_/*E* are reported in Figures S26 and S27, respectively. According to the results of the effective spin Hamiltonian modeling, the presence of simultaneous perturbations should lead to negative values of both Δ*g*_*x*_/*E* and Δ*g*_*z*_/*E*. By cross-checking the impact of each parameter (Figures S26 and S27), it is evident that only the inclusion of a negative Δ*B*_4_^3^ value can satisfy this requirement. We then simulated the experimental spectra with the inclusion of both Δ*B*_4_^4^ and Δ*B*_4_^3^ terms, which lead to the best fit parameters Δ*B*_4_^4^ = (−4.6 ± 0.3) × 10^–6^ cm^–1^ and Δ*B*_4_^3^ = (1.5 ± 0.1) × 10^–5^ cm^–1^. The combination of these two values leads to a Δ*g*_*x*_/*E* value of −2.6 × 10^–9^ m/V and a Δ*g*_*z*_/*E* value of −1.7 × 10^–9^ m/V. In Figure 6, we report the resulting simulated spectra, which are in excellent agreement with the experimental data. Interestingly, from our model it emerges that the electric field is most effective in modulating out-of-diagonal CF parameters, in agreement with the report by Ardavan and co-workers,^12^ but it also emphasizes that such modulation is equally effective in symmetry allowed (*i.e.*, *B*_4_^3^) and not-allowed parameters (*i.e.*, *B*_4_^4^).

**Figure 6:**
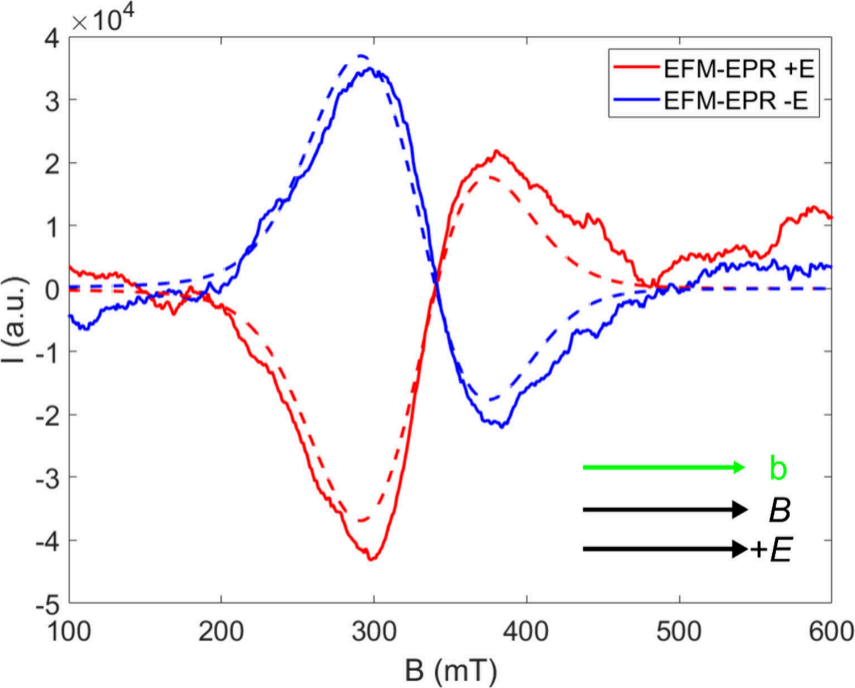
Experimental (lines) and simulated (dashed lines) EFM-EPR spectra on the Λ enantiomer acquired with electric and magnetic fields parallel to *b* crystallographic axis. Simulations are obtained considering a simultaneous modulation on the *B*_4_^4^ and *B*_4_^3^ parameters, as described in the text.

## Conclusions

In the work reported, a comprehensive characterization of the electronic structure of a mononuclear chiral dysprosium-based complex was conducted. This was achieved by combining experimental magnetometric and spectroscopic techniques with *ab initio* calculations. A comprehensive investigation was undertaken to ascertain the nature of the spin-electric effect by employing EFM-EPR. A comparison of the results obtained on the two enantiomers revealed that the topological chirality exerted no significant influence on the magnitude of the observed effects in our complex. Models of the experimental spin-electric effect of increasing complexity have been proposed to reveal different key aspects. Considering the modulation of the Landé *g*_*J*_ factor by the electric field has yielded one of the most intense effects to date. The modeling performed by considering an effective *S* = 1/2 Hamiltonian, showed that the electric field induces an anisotropic perturbation on the *g*-tensor. Such a perturbation is one order of magnitude higher in the direction perpendicular to the applied electric field than in the direction parallel to that. Finally, a more complete model incorporating a modulation of the CF parameters of the metal complex confirmed the results obtained from the effective Hamiltonian model, thus emphasizing that the electric field is most effective in modulating out-of-diagonal CF parameters. These results underline the high potential of lanthanide-based complexes to obtain remarkably strong spin electric effect along specific directions.

## Experimental Section

### Magnetometric Measurements

The samples were investigated as powders or oriented single crystals, the enantiomeric forms of which have been determined. Temperature and field-dependent direct current (DC) magnetic measurements were conducted using a Quantum Design MPMS SQUID magnetometer. The raw data were processed to remove the contribution of the sample holder and corrected for sample diamagnetism using Pascal’s constants. Cantilever torque magnetometry experiments were carried out using a commercial Quantum Design PPMS magnetometer equipped with horizontal motor and torque magnetometry options.

### THz Measurements

A usual time domain spectroscopy (TDS) setup in transmission configuration^51^ was used to measure the absorption coefficient in the frequency range 5–100 cm^–1^ of pressed pellets of polycrystalline **Dy­(oda)**_**3**_ powder mixed with high-density polyethylene. The sample pellets were prepared at a concentration of 25% with a thickness of 1 mm and a diameter of 15 mm. THz pulse emission and detection were obtained by two photoconductive antennas excited with femtosecond optical laser pulses (λ = 780 nm, Δ*t* = 120 fs at 100 MHz). The sample temperature was controlled by a close cycle helium cryostat with a temperature stability of ±0.1 K. Optical parameters extraction was performed by a custom-developed MATLAB script.^52^ Absorption coefficient as a function of frequency at a temperature of 10 K is shown in Figure S4.

### Luminescence Measurements

The measurements were performed using a Horiba PTI QM-400 spectrometer, in combination with the continuous He flow Oxford Instruments cryostat Optistat CF. The sample was prepared by grinding the polycrystalline powders and mixing them with Fluorolube oil (Sigma-Aldrich) in a mortar. A drop of this mixture was then deposited between two quartz glasses. All measurements were performed at 5 K, using an excitation wavelength of 365 nm, an excitation bandwidth of 2 nm, a detection bandwidth of 0.5 nm, a step of 0.2 nm, and a rate of 1 nm/s.

### Fitting Procedure

CF parameters for **Dy­(oda)**_**3**_ were obtained through a fitting procedure of magnetic and luminescence data using a custom MATLAB code based on the Easyspin package,^53^ version 6.0.027, to simulate the magnetic properties, and the *fminuit* minimization routine.^54^ The function to be minimized was the error between the experimental and simulated data, evaluated as the root-mean-square relative error (RMSRE).^55^

$$f\left(x\right)=RMSRE=\sqrt{\frac{1}{n}\overset{n}{\underset{i=1}{\sum }}{\left(\frac{x_{i}-y_{i}}{y_{i}}\right)}^{2}}$$ 8

*x*_*i*_ and *y*_*i*_ represent the simulated and experimental values, respectively, while *n* is the number of points of the data set. The initial guess of the fitting procedures was taken from the CF parameters calculated from *ab initio* calculations.

### EPR and EFM-EPR Measurements

X-band EPR measurements were performed using an X-band (ν ≈ 9.4 GHz) Elexsys E500 instrument (Bruker) equipped with a microwave frequency counter. An Oxford Instruments ESR900 continuous He flow cryostat was used to achieve low temperatures. An ER4122SHQE EPR resonator (Bruker) was used for the measurements.

The EFM-EPR technique is similar to EPR spectroscopy, except that, in place of the standard magnetic field modulation, an electric field modulation is used. In fact, after applying an oscillating electric field *E*(*t*) = *E*_*m*_ cos­(*ωt*) with ω = 2π·30 kHz and taking advantage of the phase-sensitive detection, a signal appears if a linear SEE is active. The introduction of the long-wavelength *E*_*m*_ in the comparatively small X-band cavity is realized by modifying the standard EPR quartz sample holder to host two thin parallel, 1 mm distant conducting wires acting as electrodes. The experimental intensity of the applied electric field was equal to 86 kV/m. Importantly, both EPR and EFM-EPR were measured on the same sample, without any modification to its position inside the cavity, allowing a correct rescaling of the experimental signals’ intensity.

The EFM-EPR and the corresponding EPR spectra used as reference were acquired at 15 K, the lowest temperature ensuring satisfactory thermal stability, given the heat load associated with the modulating *E*_*m*_. EPR spectra were acquired with a magnetic field modulation amplitude of 4 G, a frequency of 100 kHz, a microwave power of 6.7 mW, a time constant and a conversion time of 20.48 ms for the 4096 points field sweep, resulting in an acquisition time of 84 s. EFM-EPR measurements were realized with the same setup as used for the EPR measurements, except for the modified version of the sample holder (already described in ref 13). To avoid discharge, the EPR tube hosting the electrodes and the sample was filled with He gas and sealed. The EFM-EPR spectra were acquired with 32 times higher microwave power with respect to the EPR spectra. Moreover, a time constant of 327.68 ms and a conversion time of 1310.72 ms were used for the 512 point field sweep, resulting in an acquisition time of 671 s for each spectrum. When needed, multiple acquisitions were used to increase the signal-to-noise ratio of EFM-EPR spectra. The reported signals are the sum of all of the acquisitions normalized to the number of acquisitions.

### EPR and EFM-EPR Simulations

EPR and EFM-EPR spectra were simulated using custom MATLAB scripts based on the package EasySpin,^53^ version 6.0.027. For the EPR spectra, the Hamiltonian described in eq 1 was considered together with the actual value for the magnetic field modulation amplitude (*B*_*m*_) used during the experiments. While the intensity of the first-harmonic EPR spectrum is proportional to the magnetic field modulation amplitude *B*_*m*_,^56^ the intensity of the EFM-EPR spectrum is proportional to the equivalent magnetic field modulation (*B*_*m*_^*eq*^), defined as the magnetic field modulation which would yield an EFM-EPR of the same intensity if it were acquired using *B*_*m*_.^10^ From *B*_*m*_^*eq*^ the Δ*g* induced by the electric field can be obtained by

9 $${\Delta g}_{E}=g\frac{B_{m}^{eq}}{B_{res}}$$

where *g* and *B*_*res*_ are the *g* factor and resonant field values of the EPR spectrum. Based on eq 9, a “first derivative” EFM-EPR spectrum resulting from the electric field modulation and phase-sensitive detection can be simulated as the difference between the absorption spectrum corresponding to Δ*g*_*E*_(+*E*_*m*_) and that corresponding to Δ*g*_*E*_(−*E*_*m*_). From this procedure, the value of Δ*g*_*E*_(*E*_*m*_) can be estimated. Similarly, a “first derivative” EPR spectrum can be simulated as the difference between the absorption spectrum corresponding to Δ*g*_*E*_(+*B*_*m*_) and that corresponding to Δ*g*_*E*_(−*B*_*m*_).

### *Ab Initio* Calculations

The isolated structure of the Λ enantiomer of **Dy­(oda)**_**3**_ was optimized in the gas phase with G09 quantum chemistry software. The geometry relaxation was performed using the PBE0 ^57^ functional and the LANL2DZ basis set,^58^ starting from the crystallographic structure and maintaining the *C*_3_ point group during optimization. For the relaxed structure, multiconfigurational Complete Active Space Self Consistent Field (CASSCF) calculations were performed using the ORCA 6.0.1 ^59^ software, which allows the inclusion of an electric field of arbitrary direction and magnitude during the orbital optimization. Scalar relativistic effects were included within the Zero-Order Relativistic Approximation (ZORA). SARC2-ZORA-QZVP^60^ basis sets were employed for the lanthanide ion, ZORA-def2-TZVP^61^ for the oxygens in the first coordination sphere and ZORA-def2-SVP for the remaining atoms. “Defgrid3” integration accuracy within the RIJCOSX approximation^62^ was employed. The active space consisted of the 9 electrons in the *4f* orbitals of the lanthanide ion, CAS­(9,7). 21 sextuplets, 224 quadruplets, and 130 doublets were computed and included in the subsequent state interaction calculation (CASSI). The CF parameters were mapped within the Extended Stevens’ Operators (ESO) formalism through the SINGLE_ANISO formalism^63,64^ as implemented in Orca. The convergence criteria for all of the CASSCF calculations have been set to 1 × 10^–10^ Ha. We conducted additional calculations with tighter SCF and geometry optimization convergence thresholds (down to 1 × 10^–12^ Ha in energy). These more stringent criteria yielded consistent Δ*B*_*k*_^*q*^ values, confirming that the reported trends are not artifacts of numerical noise.

## Supplementary Material



## Notes

Additional figures and tables as described in the text (PDF)
