# Supplementary material for: Spin-Electric Effect on a Chiral Dysprosium Complex
Source: J Am Chem Soc. 2025 Aug 26;147(36):33041–52. doi: 10.1021/jacs.5c10840 (PMC12426923; doi:10.1021/jacs.5c10840)
Supplement: Supplementary file 1 [file ja5c10840_si_001.pdf]

Supporting Information

Spin-electric effect on a chiral dysprosium complex

Leonardo Tacconi[a], Alberto Cini[b], Arsen Raza[a,c], Lorenzo Tesi[d], Paolo Bartolini[e], Andrea Taschin[e,f], Joris van Slageren[d], Matteo Briganti[a]*, Lorenzo Sorace[a]*, Maria Fittipaldi[b]*, Mauro Perfetti[a]*

a. Department of Chemistry "Ugo Schiff", University of Florence and INSTM Research Unit, Via della Lastruccia 3-13, 50019 Sesto Fiorentino (FI), Italy.

b. Department of Physics and Astronomy, University of Florence and INSTM Research Unit, Via Sansone 1, Sesto Fiorentino (FI), Italy.

c. Department of Industrial Engineering, DIEF, 50139 Florence, Italy.

d. Institute of Physical Chemistry and Center for Integrated Quantum Science and Technology, University of Stuttgart, Pfaffenwaldring 55, 70569, Stuttgart, Germany.

e. Consiglio Nazionale delle Ricerche - Istituto Nazionale di Ottica, CNR-INO, Via Nello Carrara 1, Sesto Fiorentino FI, 50019, Italy.

f. European Lab. for Non-Linear Spectroscopy (LENS), Univ. di Firenze, via N. Carrara 1, I-50019 Sesto Fiorentino, Firenze, Italy.

Supporting Tables

Table S1 – Crystallographic data and refinement details for **Y(oda)$_3$**.

**Compound reference**	**Y(oda)$_3$**
Chemical formula	$C_{12}H_{24}B_2F_8Na_5O_{21}Y$
Formula mass	881.79
Crystal system	Hexagonal
a/Å	9.5859(6)
b/Å	9.5859(6)
c/Å	27.885(3)
α/°	90
β/°	90
γ/°	120
Unit cell volume/Å^3	2219.0(3)
Temperature/K	100.0(1)
Space group	*R32*
No. of formula units/unit cell, *Z*	3
Radiation type	Mo Kα
Absorption coefficient, μ/mm^{-1}	2.182
No. of reflections measured	13432
No. of independent reflections	1498
Data/restraints/parameters	1498/0/88
Final R_1 values (all data)	0.0366
Final $wR_2(F^2)$ values (all data)	0.0933
Final R_1 values ($I > 2\sigma(I)$)	0.0355
Final $wR_2(F^2)$ values ($I > 2\sigma(I)$)	0.0940
Goodness of fit on F^2	1.062
Largest diff. peak and hole (eÅ^{-3})	0.837/-1.687
CCDC number	2476962

Table S2 – Crystal field parameters of **Dy(oda)$_3$** obtained from *ab initio* calculations and expressed in cm^{-1} in Stevens' notation. The relevant ones in the considered symmetry are evidenced in bold.

B_2^2	-7.537E-05	B_4^0	**4.124E-03**	B_6^2	2.507E-09
B_2^1	2.987E-05	B_4^{-1}	-1.280E-09	B_6^1	4.687E-09
B_2^0	**-7.991E-02**	B_4^{-2}	6.352E-10	B_6^0	**-5.936E-06**
B_2^{-1}	6.444E-07	B_4^{-3}	1.674E-08	B_6^{-1}	1.650E-11
B_2^{-2}	-2.174E-07	B_4^{-4}	6.488E-10	B_6^{-2}	1.498E-11
B_4^4	6.962E-07	B_6^6	**-4.074E-04**	B_6^{-3}	7.517E-11
B_4^3	**6.513E-02**	B_6^5	-2.223E-08	B_6^{-4}	-2.262E-11
B_4^2	-2.608E-07	B_6^4	-6.178E-09	B_6^{-5}	2.648E-10
B_4^1	4.979E-07	B_6^3	**-4.044E-04**	B_6^{-6}	-1.218E-11

Table S3 – Energy structure of the Kramers doublets (KD) of **Dy(oda)$_3$** in zero magnetic field, Boltzmann population of the levels at 5K, calculated from the crystal field parameters obtained through *ab initio* calculations as described in main, and main values of the g-tensor. Only m_J with a percentage above 5% were shown in the composition column.

**KD**	$E\ (cm^{-1})$	**Composition %**	**Boltzmann population at 5 K**	***g* tensor components (g_x, g_y, g_z)**
1	0	$77.4\%\vert\pm 9/2>$<br>$15.6\%\vert\pm 3/2>$<br>$6.7\%\vert\mp 3/2>$	0.706	0.001 0.003 9.6353
2	3.09	$64.5\%\vert\pm 11/2>$<br>$26.9\%\vert\pm 5/2>$<br>$5.0\%\vert\mp 1/2>$	0.290	1.314 1.314 10.798
3	18.4	$12.5\%\vert\pm 13/2>$<br>$64.7\%\vert\pm 7/2>$<br>$14.8\%\vert\pm 1/2>$	0.004	0.459 0.460 7.379
4	79.1	$80.6\%\vert\pm 13/2>$<br>$8.7\%\vert\pm 7/2>$<br>$16.1\%\vert\mp 11/2>$	0.000	4.430 4.430 13.408
5	121.6	$6.1\%\vert\pm 13/2>$<br>$4.1\%\vert\pm 7/2>$<br>$17.6\%\vert\pm 1/2>$<br>$46.9\vert\mp 5/2>$<br>$25.3\vert\mp 11/2>$	0.000	6.523 6.521 4.752
6	140.4	$82.3\%\vert\pm 15/2>$<br>$13.1\%\vert\pm 3/2>$	0.000	0.000 0.000 17.218
7	174.4	$17.1\%\vert\pm 15/2>$<br>$16.8\%\vert\pm 9/2>$<br>$60.2\%\vert\pm 3/2>$	0.000	0.001 0.002 7.310
8	202.5	$19.0\%\vert\pm 7/2>$<br>$62.4\%\vert\pm 1/2>$<br>$17.6\%\vert\mp 5/2>$	0.000	10.291 10.288 1.424

Table S4 – Comparison between the crystal field parameters of **Dy(oda)$_3$** obtained through the fitting procedure described in main, ab initio calculations and parameters reported in literature obtained via spectroscopy and ab initio calculations. Parameters are expressed in cm^{-1} and in Wybourne's notation[1] to ease their comparison.

	**Fitting procedure**	**Ab initio calculations**	**Metcalf et al.**	**Baldovì et al.**	**Fitting procedure with distortion**
B_2^0	72	25	59	22	36
B_4^0	-876	-557	-757	-927	-796
B_6^0	-219	-92	-392	-565	-131
B_4^3	-556	372	588	474	468
B_6^3	-448	305	620	446	436
B_6^6	-627	-414	-581	-585	-572
B_2^2	/	/	/	/	-24

Table S5 – Energy structure of the Kramers doublets (KD) of **Dy(oda)$_3$** in zero magnetic field and Boltzmann population at 5K calculated using the crystal field parameters determined by the global fitting procedure described in main. Only m_J with a percentage above 5% were shown in the composition column.

KD	$E\ (cm^{-1})$	Composition %	Boltzmann population at 5 K
1	0	$79.7\%\|\pm 9/2>$<br>$13.6\%\|\pm 3/2>$<br>$6.4\%\|\mp 3/2>$	0.863
2	6.40	$63.6\%\|\pm 11/2>$<br>$26.8\%\|\pm 5/2>$<br>$6.1\%\|\mp 7/2>$	0.137
3	30.2	$11.8\%\|\pm 13/2>$<br>$64.0\%\|\pm 7/2>$<br>$13.9\%\|\pm 1/2>$<br>$7.3\%\|\mp 11/2>$	0.000
4	120.9	$81.7\%\|\pm 13/2>$<br>$7.8\%\|\pm 7/2>$<br>$5.8\%\|\mp 11/2>$	0.000
5	190.5	$22.9\%\|\pm 11/2>$<br>$50.2\%\|\pm 5/2>$<br>$15.6\%\|\mp 1/2>$<br>$5.5\%\|\mp 7/2>$<br>$5.7\%\|\mp 13/2>$	0.000
6	205.0	$89.9\%\|\pm 15/2>$<br>$7.9\%\|\pm 3/2>$	0.000
7	269.3	$9.9\%\|\pm 15/2>$<br>$16.0\%\|\pm 9/2>$<br>$67.7\%\|\pm 3/2>$	0.000
8	314.4	$16.6\%\|\pm 7/2>$<br>$66.8\%\|\pm 1/2>$<br>$15.4\%\|\mp 5/2>$	0.000

Table S6 - Energy structure of the Kramers doublets (KD) of **Dy(oda)₃** in zero magnetic field and Boltzmann population of the levels at 5K calculated from the crystal field parameters reported in literature and obtained through spectroscopic studies[2]. Only m_J with a percentage above 5% were shown in the composition column.

KD	$E\ (cm^{-1})$	Composition %	Boltzmann population at 5 K
1	0	$77.2\% \vert \pm 9/2 >$<br>$16.6\% \vert \pm 3/2 >$<br>$4.9\% \vert \mp 3/2 >$	0.999
2	21.7	$51.3\% \vert \pm 11/2 >$<br>$35.4\% \vert \pm 5/2 >$<br>$10.9\% \vert \mp 7/2 >$	0.001
3	33.7	$10.2\% \vert \pm 11/2 >$<br>$19.2\% \vert \mp 1/2 >$<br>$66.4\% \vert \mp 7/2 >$	0.000
4	149.6	$87.0\% \vert \pm 13/2 >$<br>$6.1\% \vert \mp 13/2 >$	0.000
5	192.1	$8.0\% \vert \pm 13/2 >$<br>$11.1\% \vert \pm 1/2 >$<br>$46.5 \vert \mp 5/2 >$<br>$32.1 \vert \mp 11/2 >$	0.000
6	194.4	$89.8\% \vert \pm 15/2 >$<br>$8.6\% \vert \pm 3/2 >$	0.000
7	284.5	$9.1\% \vert \pm 15/2 >$<br>$20.8\% \vert \pm 9/2 >$<br>$64.5\% \vert \pm 3/2 >$	0.000
8	336.7	$17.4\% \vert \pm 7/2 >$<br>$66.3\% \vert \pm 1/2 >$<br>$15.0\% \vert \mp 5/2 >$	0.000

Table S7 – Energy structure of the Kramers doublets (KD) of **Dy(oda)$_3$** in zero magnetic field and Boltzmann population of the levels at 5K calculated from the crystal field parameters reported in literature and obtained through ab initio calculations.[3] Only m_J with a percentage above 5% were shown in the composition column.

**KD**	$E\ (cm^{-1})$	**Composition %**	**Boltzmann population at 5 K**
1	0	$84.3\%\|\pm 9/2>$<br>$10.1\%\|\pm 3/2>$<br>$5.2\%\|\mp 3/2>$	0.994
2	17.7	$15.6\%\|\pm 11/2>$<br>$27.6\%\|\pm 5/2>$<br>$55.7\%\|\mp 7/2>$	0.006
3	42.4	$54.3\%\|\pm 11/2>$<br>$5.1\%\|\pm 5/2>$<br>$13.3\%\|\mp 1/2>$<br>$25.4\%\|\mp 7/2>$	0.000
4	164.6	$68.4\%\|\pm 13/2>$<br>$14.3\%\|\mp 5/2>$<br>$12.7\%\|\mp 11/2>$	0.000
5	191.4	$27.0\%\|\pm 13/2>$<br>$8.4\%\|\pm 7/2>$<br>$44.0\%\|\mp 5/2>$<br>$16.4\|\mp 11/2>$	0.000
6	218.9	$84.6\%\|\pm 15/2>$<br>$13.0\%\|\pm 3/2>$	0.000
7	273.7	$15.1\%\|\pm 15/2>$<br>$12.1\%\|\pm 9/2>$<br>$67.5\%\|\pm 3/2>$	0.000
8	326.4	$10.3\%\|\pm 7/2>$<br>$77.7\%\|\pm 1/2>$<br>$9.0\%\|\mp 5/2>$	0.000

Table S8 - Values of the coefficient of determination R^2 obtained by fitting the variations of the crystal field parameters ΔB_k^q as a function of applied electric field, determined by *ab initio* calculations, with a linear function. Bold characters evidence the parameters for which linear correlation is observed.

ΔB_2^2	**0.8151**	ΔB_4^0	0.0233	ΔB_6^2	**0.9527**
ΔB_2^1	**0.9925**	ΔB_4^{-1}	0.0294	ΔB_6^1	0.0049
ΔB_2^0	0.2364	ΔB_4^{-2}	0.1905	ΔB_6^0	0.0205
ΔB_2^{-1}	0.0183	ΔB_4^{-3}	0.0064	ΔB_6^{-1}	0.2732
ΔB_2^{-2}	0.4106	ΔB_4^{-4}	0.0506	ΔB_6^{-2}	0.4593
ΔB_4^4	**0.9215**	ΔB_6^6	0.1006	ΔB_6^{-3}	0.0391
ΔB_4^3	0.0826	ΔB_6^5	**0.9076**	ΔB_6^{-4}	0.0015
ΔB_4^2	**0.9823**	ΔB_6^4	**0.9958**	ΔB_6^{-5}	0.0060
ΔB_4^1	0.2563	ΔB_6^3	0.1522	ΔB_6^{-6}	0.0361

Supporting Figures

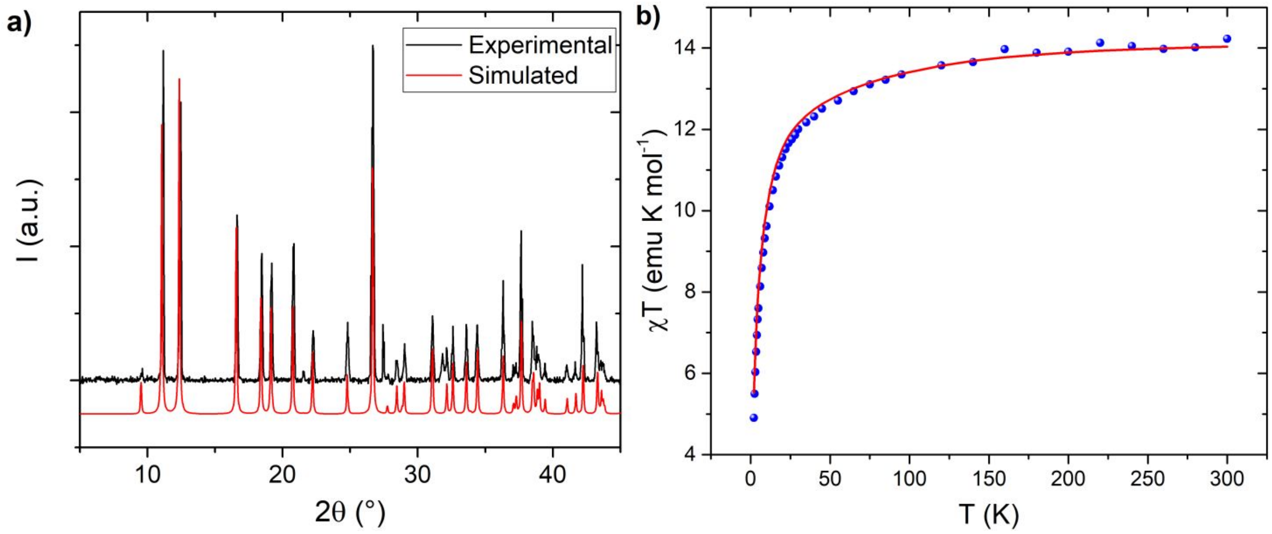

Figure S1 – (**a**) Experimental PXRD pattern acquired on polycrystalline **Dy(oda)**$_3$ compared to the one simulated from its crystal structure. (**b**) χT vs T curve acquired on polycrystalline **Dy(oda)**$_3$ in an applied magnetic field B = 100 mT. Blue dots represent the experimental points while red line is the curve simulated using the Hamiltonian operator and best fit parameters discussed in main text.

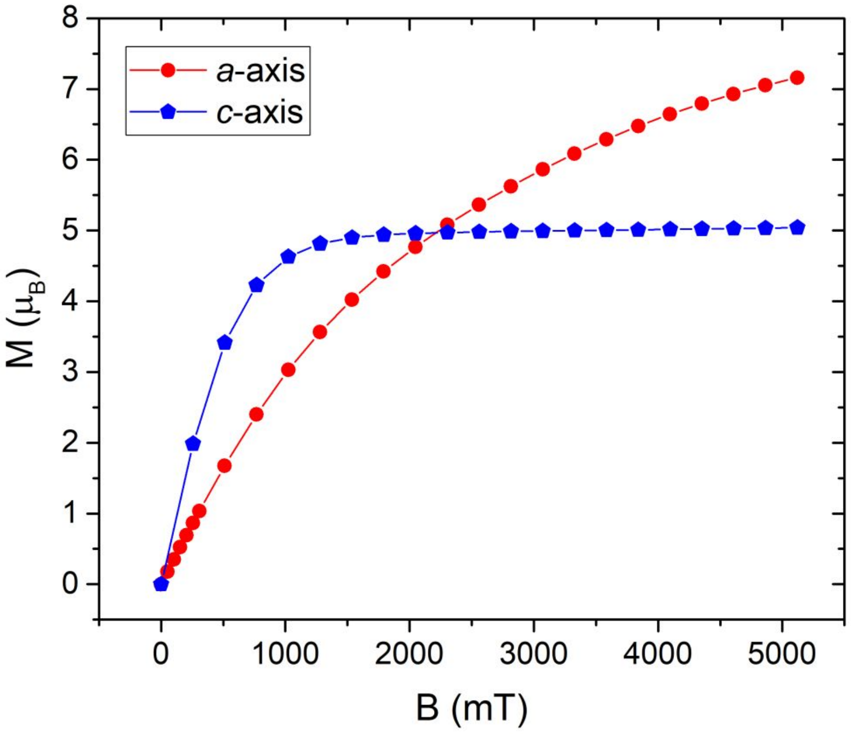

Figure S2 – Experimental M vs B curves of **Dy(oda)**$_3$ at T = 2 K, along two distinct crystallographic orientations. The intersection of the two curves above 2000 mT indicates that by increasing the magnetic field the anisotropy of the complex switches from easy-axis to easy-plane. Lines are guide to the eye.

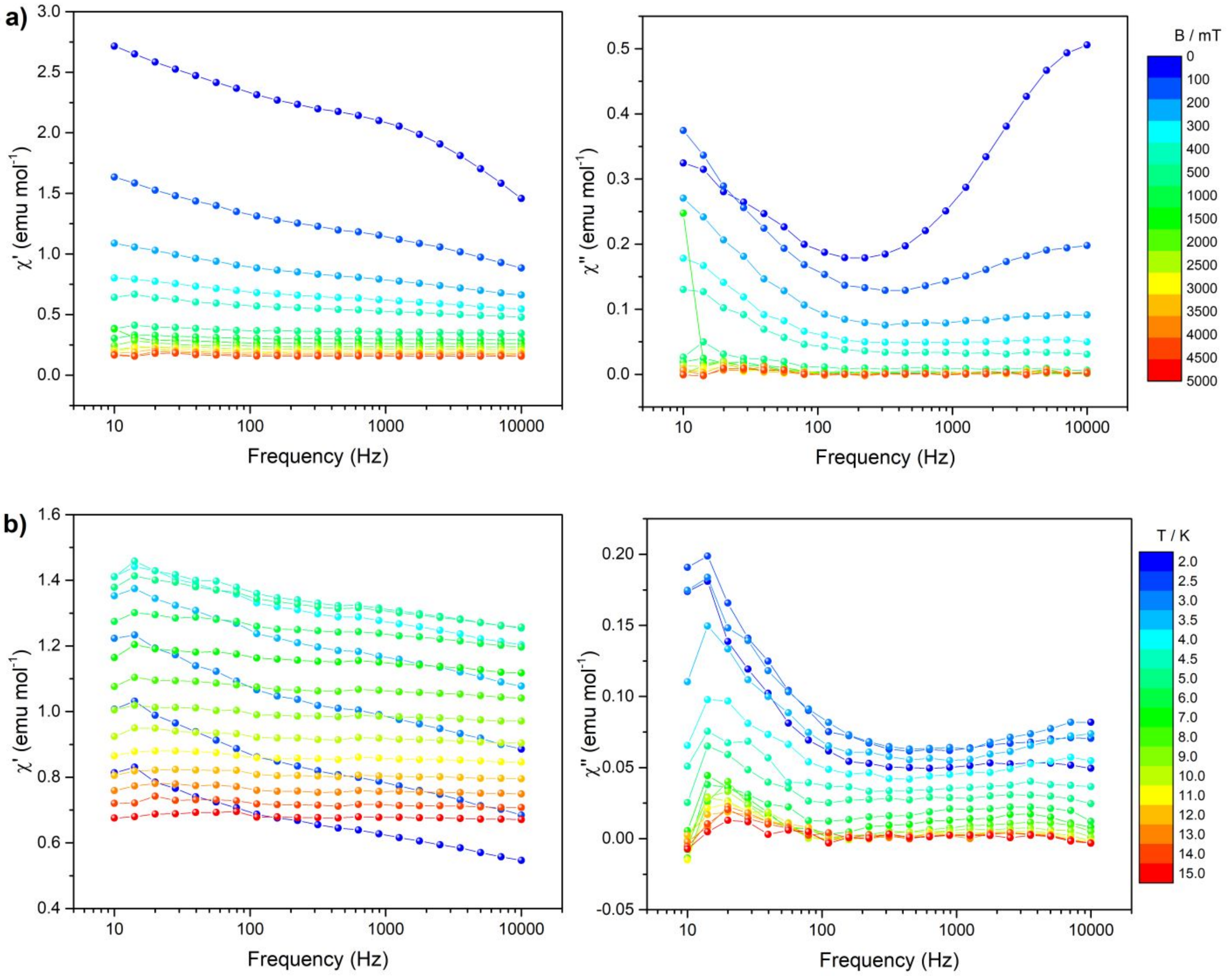

Figure S3 – Frequency dependence of Alternate Current (AC) measurements performed on a polycrystalline sample of **Dy(oda)$_3$** at 2 K as a function of applied static magnetic field (**a**) and at 100 mT as a function of temperature (**b**). Lines are guide to the eye.

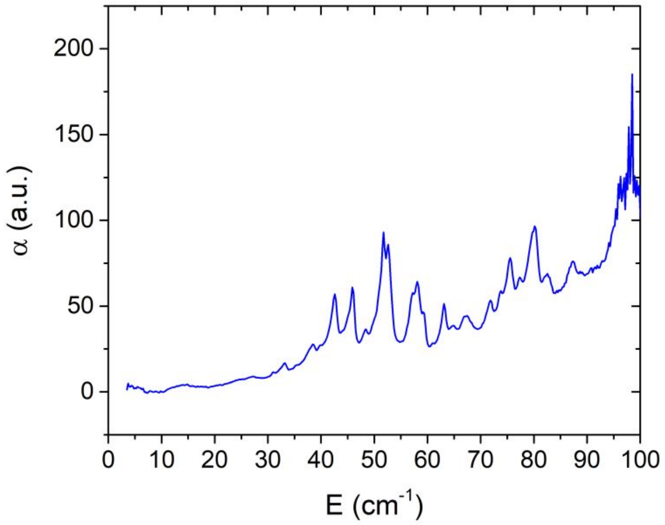

Figure S4 – THz spectrum acquired at 10K reporting the absorbance of a pressed pellet of polycrystalline **Dy(oda)$_3$**.

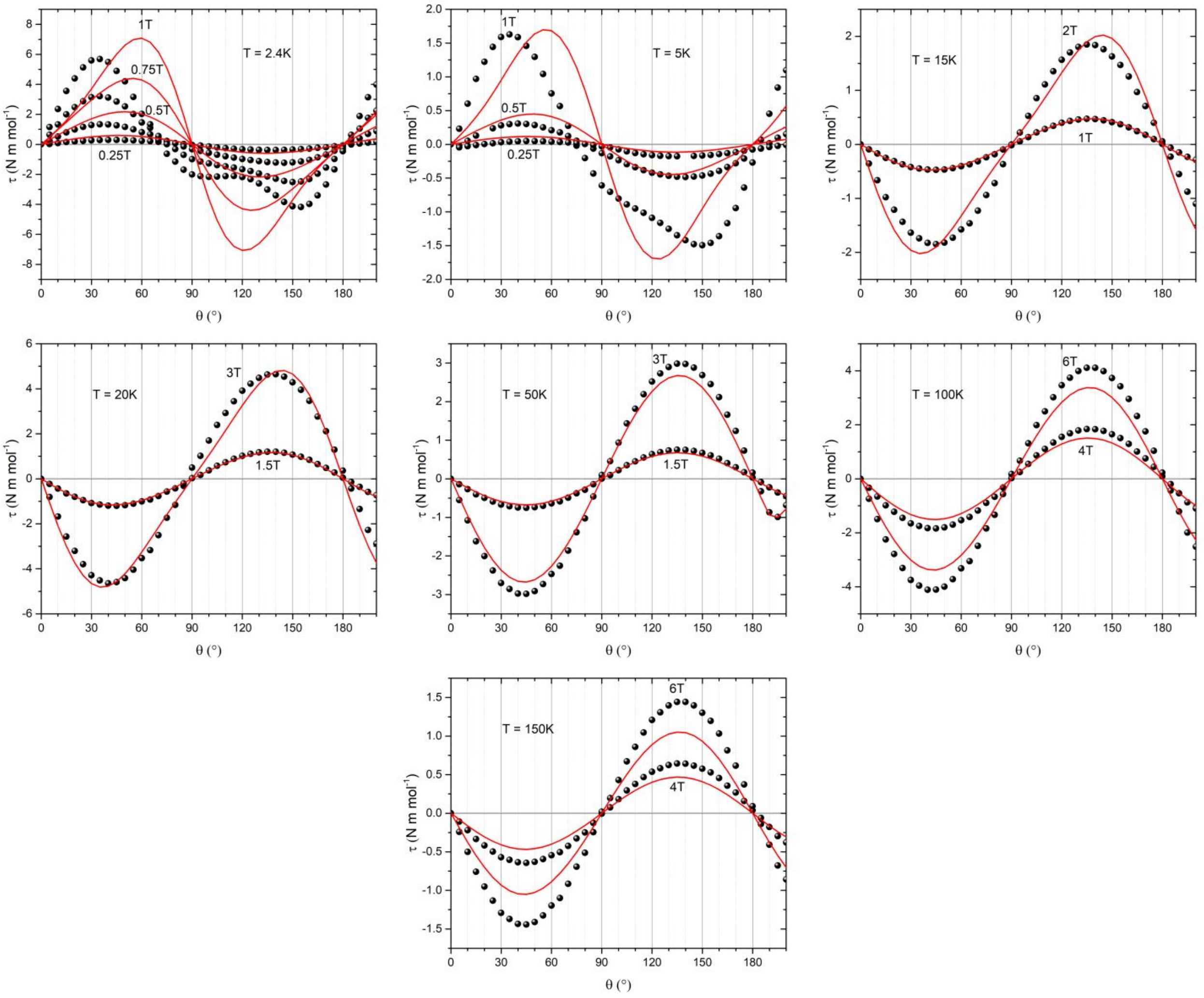

Figure S5 – Cantilever Torque Magnetometry (CTM) curves of **Dy(oda)$_3$** at several temperatures and magnetic fields. Black dots are the experimental data, while red lines represent the simulated data using the Hamiltonian parameters obtained with the global fitting procedure discussed in main. $\theta = 0°$ and $\theta = 90°$ represent the crystallographic *c* and *a* axes, respectively. At 15 K and above the shapes of the experimental results suggest that the system is axial, *i.e*. the C_3 axis is the z magnetic axis.

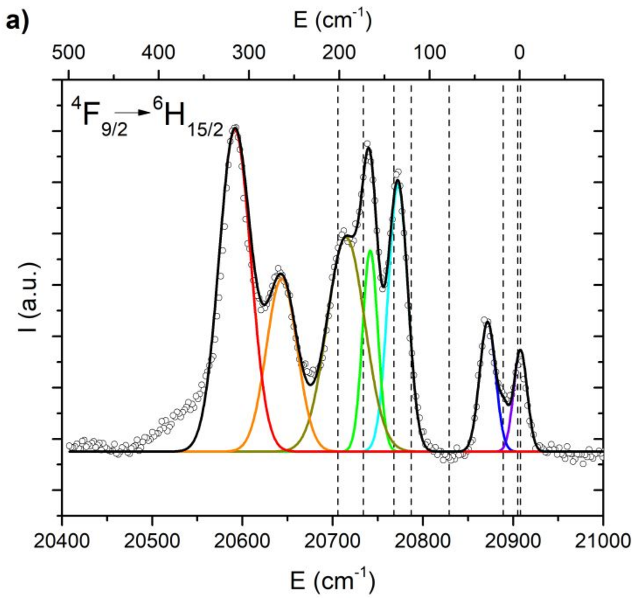

Figure S6 – Luminescence spectrum acquired on polycrystalline **Dy(oda)$_3$** at 5K, λ_{exc} = 365 nm. The experimental spectrum was fitted with 7 Gaussian curves, corresponding to the 7 most intense peaks. The coloured lines correspond to the single Gaussian curves while the black curve corresponds to the sum. The dashed lines correspond to the crystal field energies obtained from (**a**) ab initio calculations ;(**b**) the best fit of the experiments (magnetism and luminescence, see Table S2). Bottom x-scale indicates the luminescence energies for the $^4F_{9/2} \rightarrow {}^6H_{15/2}$ transition, while top x-scale indicates the crystal field splitting on the $^6H_{15/2}$ ground state.

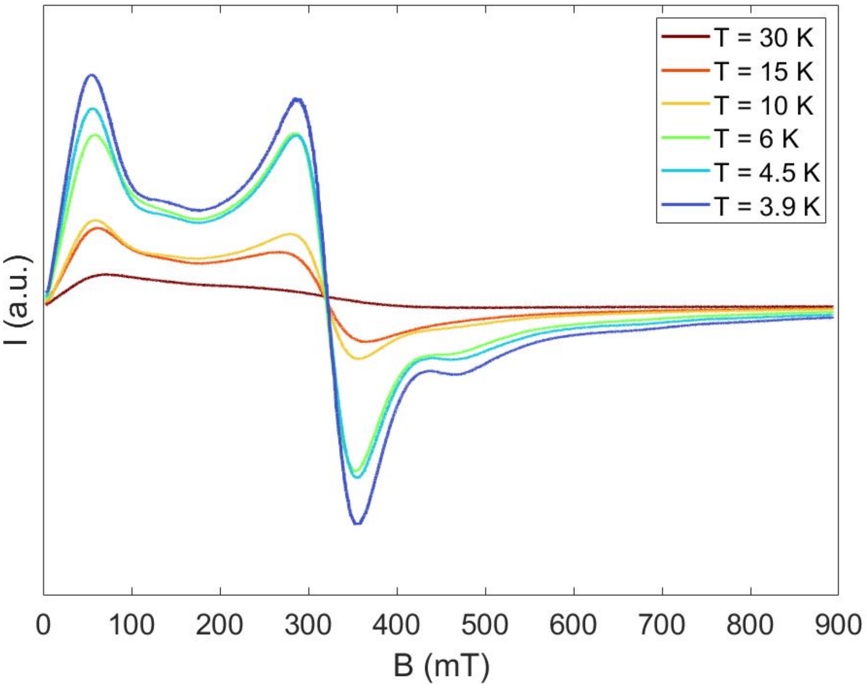

Figure S7 – X-band ($\mathcal{V}$ = 9.402 GHz) EPR spectra acquired on polycrystalline **Dy(oda)$_3$** at different temperatures.

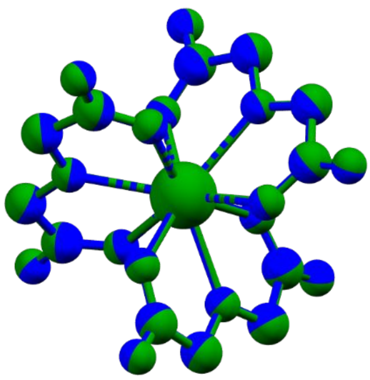

Figure S8 – Structure overlay of **Dy(oda)$_3$** (green) and **Y(oda)$_3$** (blue). The average distance between the atoms of the two structures is 0.017 Å.

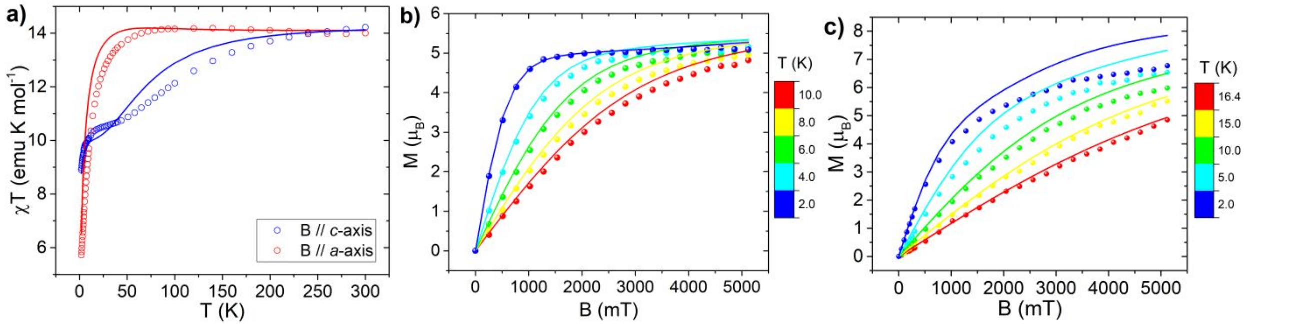

Figure S9 – Experimental (dots) and simulated (lines) DC measurements on single crystals of **Dy(oda)$_3$**. Simulations were done using the crystal field parameters obtained via *ab initio* calculations, as discussed in main text.

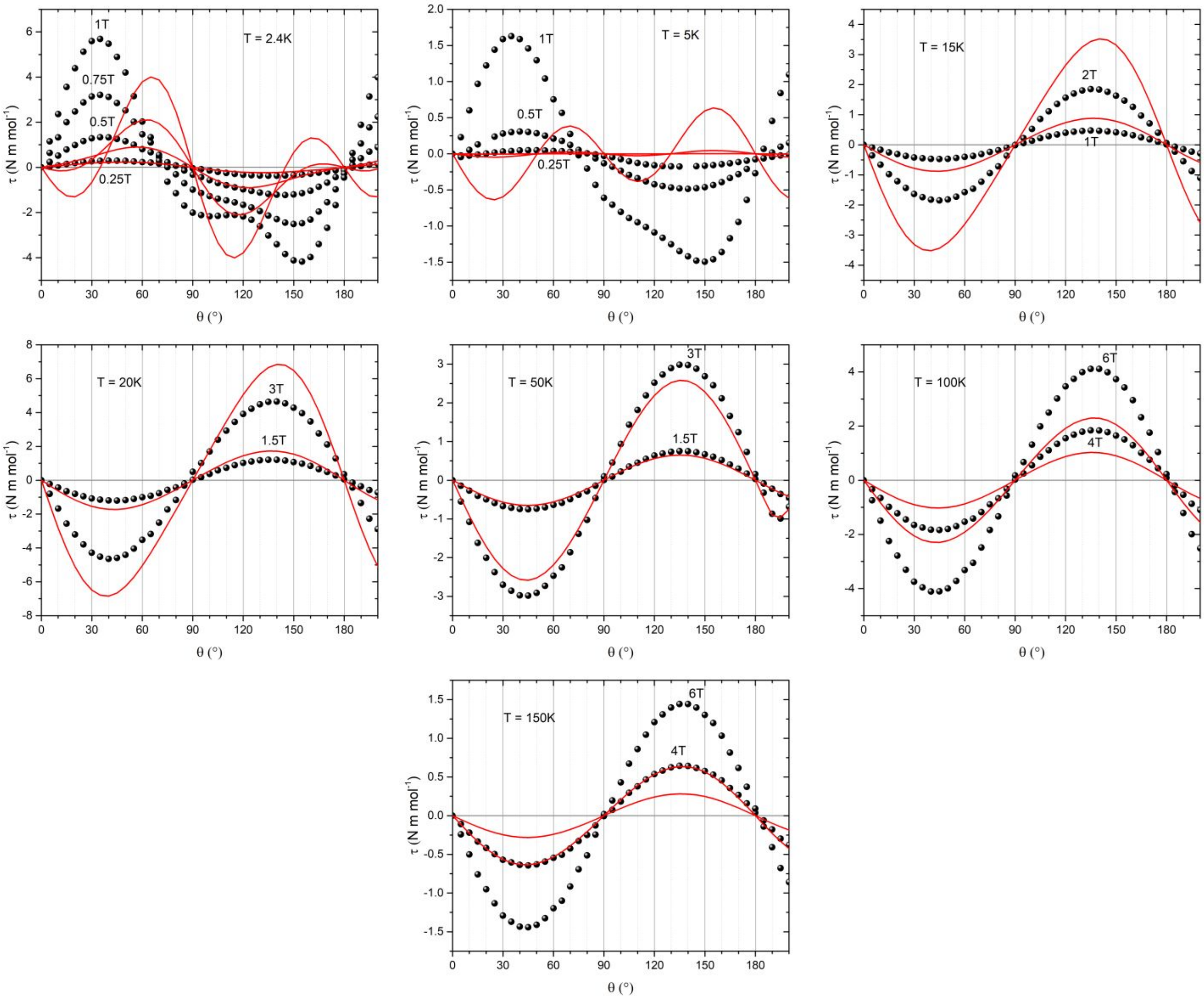

Figure S10 – Cantilever Torque Magnetometry (CTM) curves at several temperatures and magnetic fields. Black dots are the experimental data, while red lines represent the simulated data using the crystal field parameters obtained with *ab initio* calculations, as discussed in main text. $\theta = 0°$ and $\theta = 90°$ represent the crystallographic *c* and *a* axes, respectively.

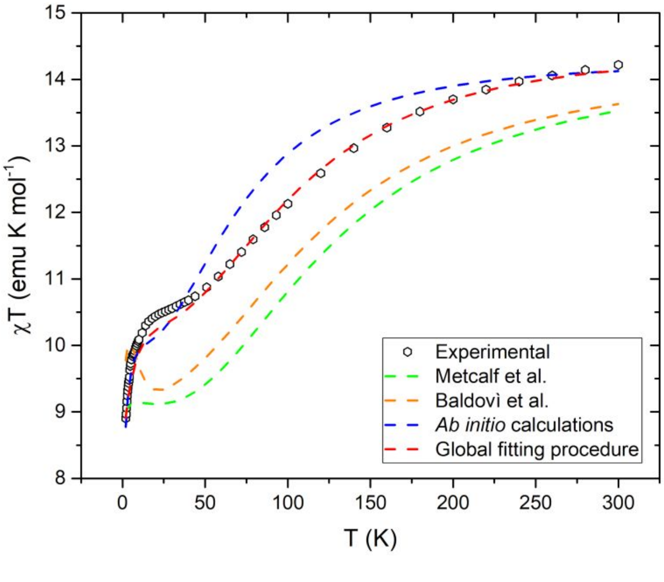

Figure S11 – Comparison between the experimental χT vs T curve acquired along the crystallographic c-axis at 100 mT and the simulated ones using different sets of crystal field parameters, as discussed in main.

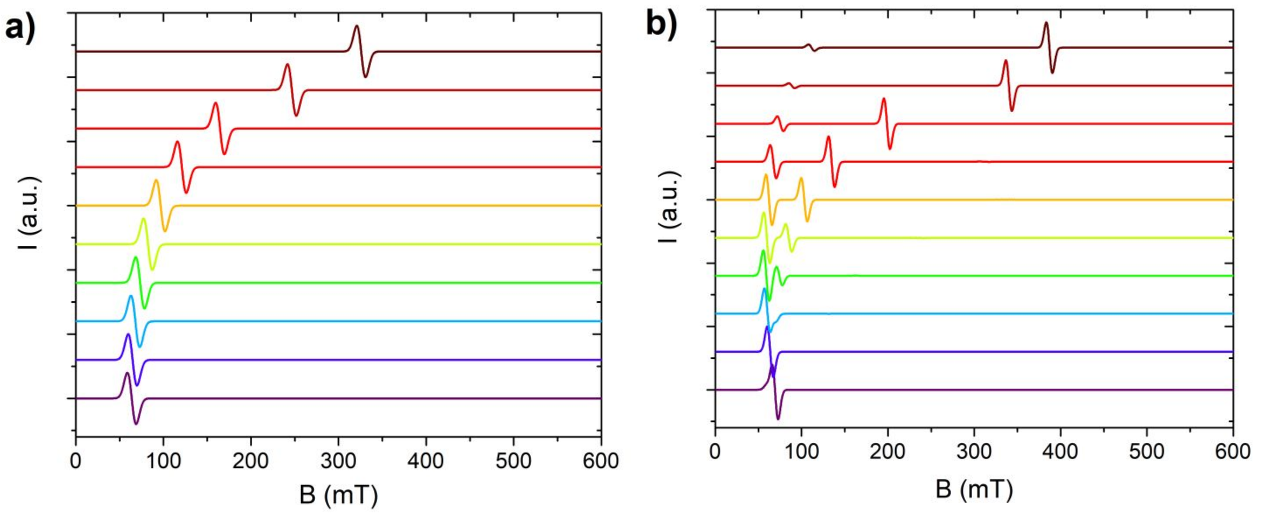

Figure S12 – Angular dependent EPR spectra simulated at 5K considering a strictly trigonal (**a**) and a slightly distorted (**b**) crystal field, as discussed in text.

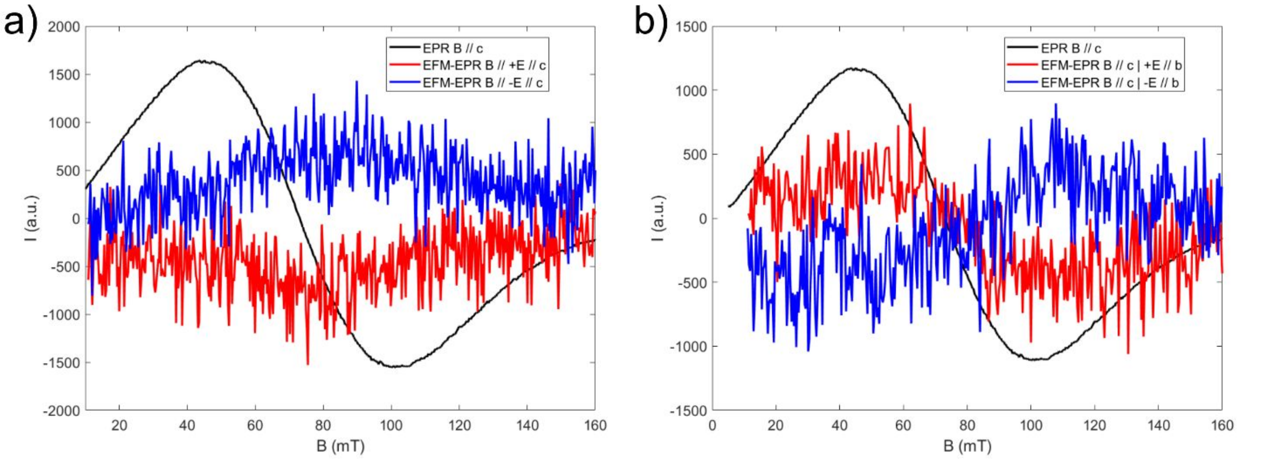

Figure S13 – EFM-EPR spectra acquired at 15 K considering various combination of orientations for the applied electric and magnetic fields on the Δ enantiomer: both parallel to the *c* axis (**a**, with 8 (blue line) and 8 (red line) acquisitions); ***B*** parallel to the *c* axis and ***E*** parallel to the *b* axis (**b**, with 9 (blue line) and 7 (red line) acquisitions). The corresponding EPR spectrum is also shown (black line), appropriately rescaled to facilitate the comparison with the EFM-EPR spectra.

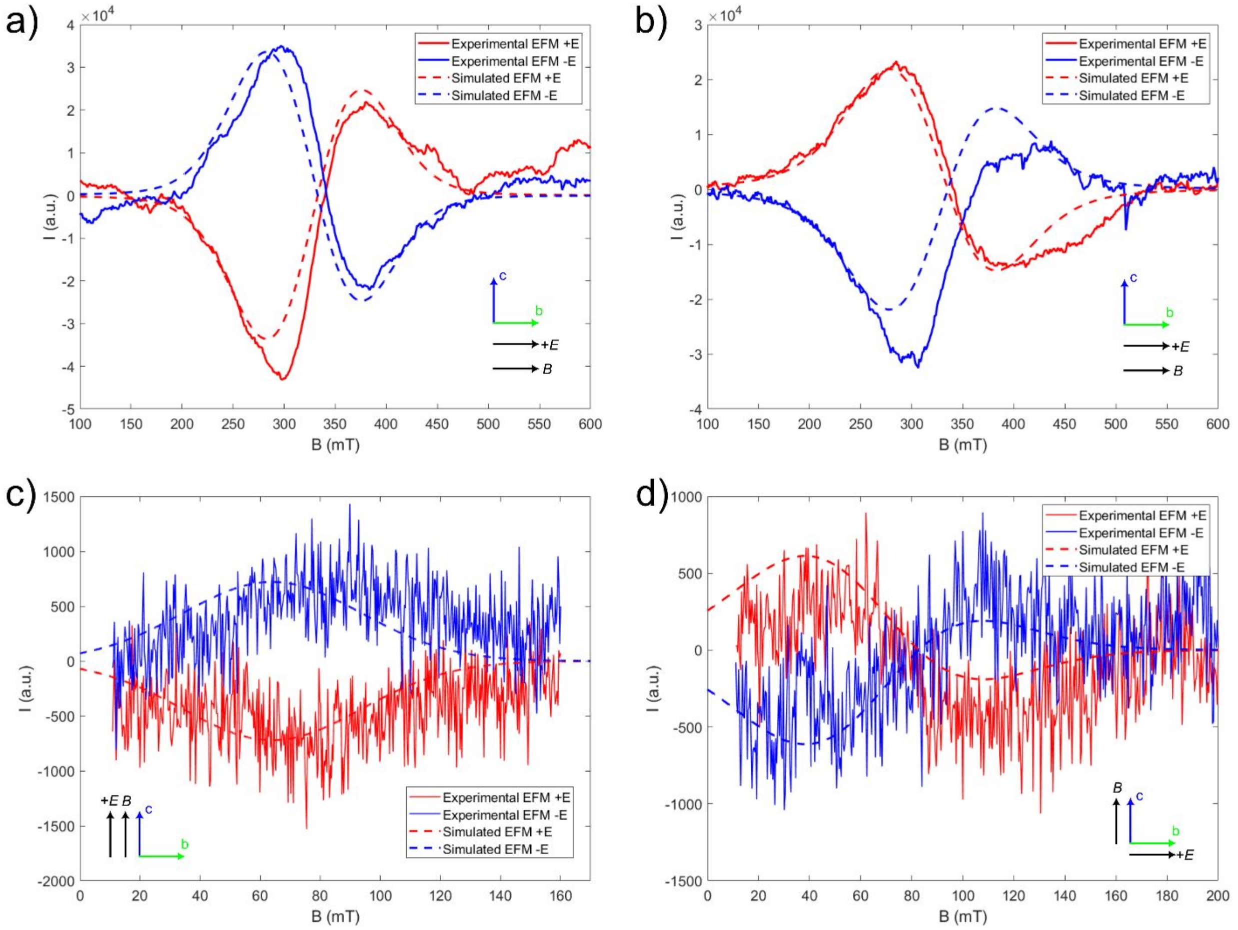

Figure S14 – Experimental (continuous lines) and simulated (dashed lines) EFM-EPR spectra of **Dy(oda)$_3$** with various combination of orientations for the applied electric and magnetic fields: both parallel to the *b* axis on the Λ enantiomer (**a**); both parallel to the *b* axis on the Δ enantiomer (**b**); both parallel to the c axis on the Δ enantiomer (**c**); ***B*** parallel to the c axis and ***E*** parallel to the b axis (**d**). Simulations were done considering a modulation of the g_J factor by the applied electric field, as discussed in main text.

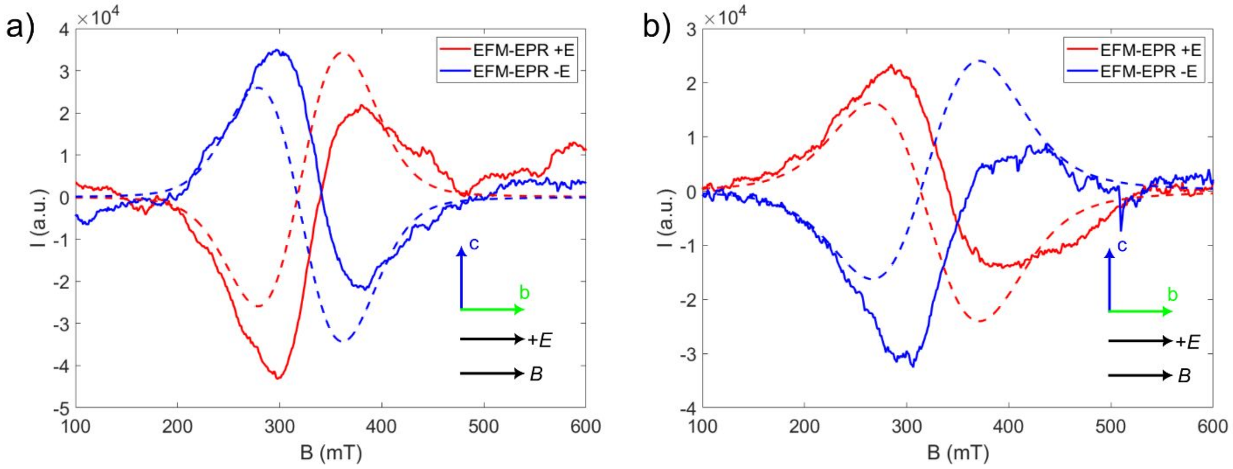

Figure S15 – Experimental (continuous lines) and simulated (dashed lines) EFM-EPR spectra of **Dy(oda)$_3$** with different combination of orientations for the applied electric and magnetic fields: both parallel to the *b* axis on the Λ enantiomer (**a**); both parallel to the *b* axis on the Δ enantiomer (**b**). Simulations were done considering a modulation by the applied electric field of the g_x factor of an effective spin 1/2 Hamiltonian mimicking the first excite state doublet, as discussed in main text.

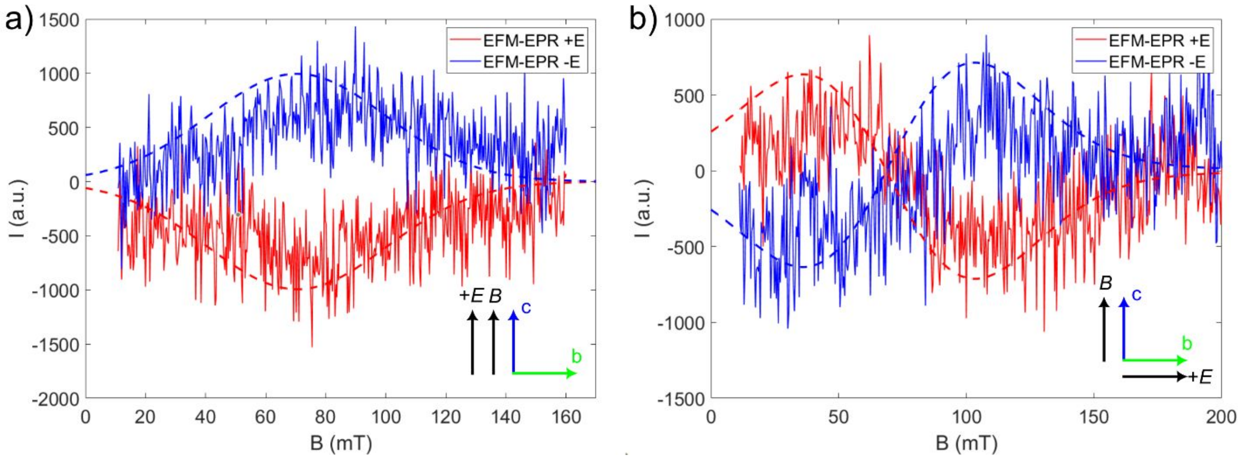

Figure S16 – Experimental (lines) and simulated (dashed lines) EFM-EPR spectra of Δ enantiomer of **Dy(oda)$_3$** with various combination of orientations for the applied electric and magnetic fields: both parallel to the *c* axis (**a**); ***B*** parallel to the c axis and ***E*** parallel to the *b* axis (**b**). Simulations were done considering a modulation by the applied electric field of the ***g*** factor of an effective spin 1/2 Hamiltonian mimicking the ground state doublet, as discussed in main text.

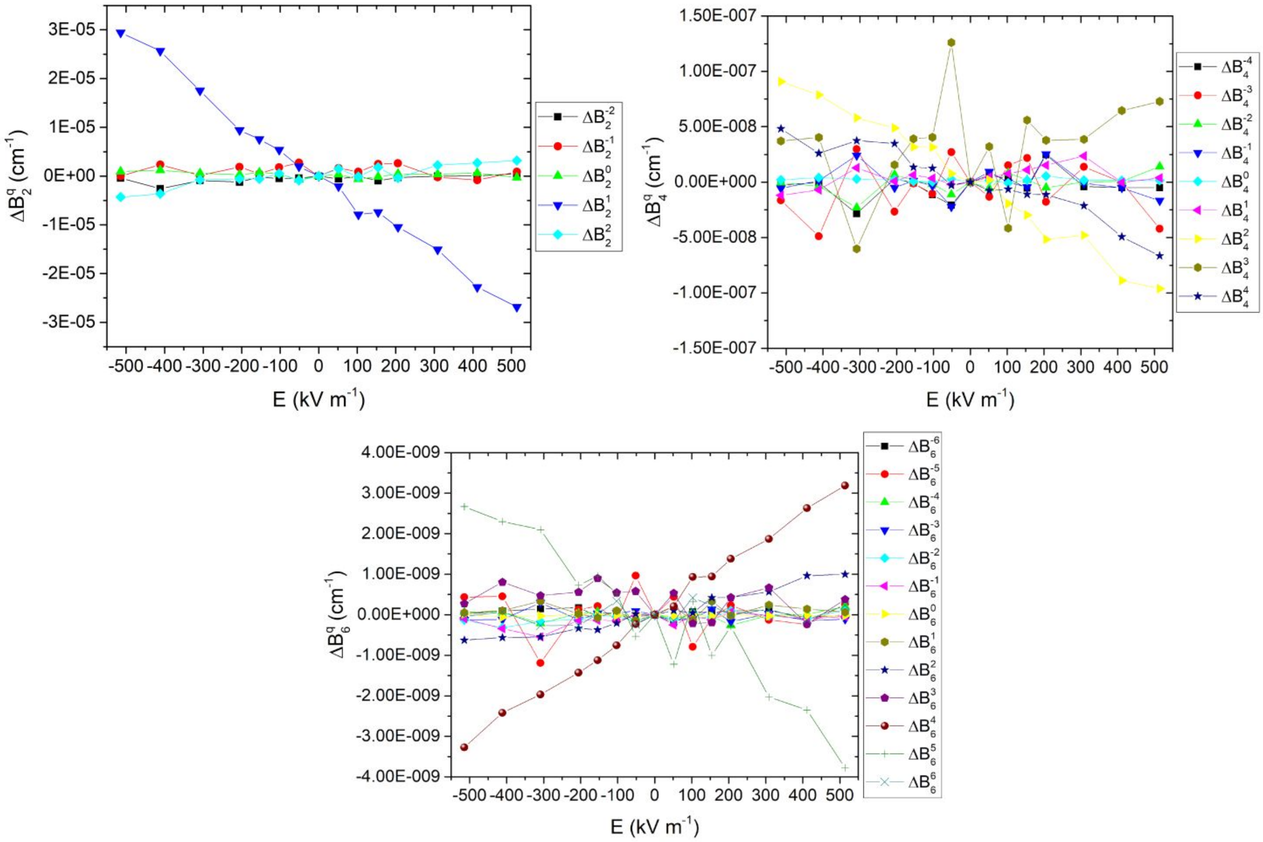

Figure S17 – Variation of the crystal field parameters (ΔB_k^q) as a function of the intensity of applied electric field, determined by *ab initio* calculations on the Λ isomer. Calculations done considering the perturbation on the electronic cloud induced by the applied electric field. Positive electric field is applied along the crystallographic -*b* axis.

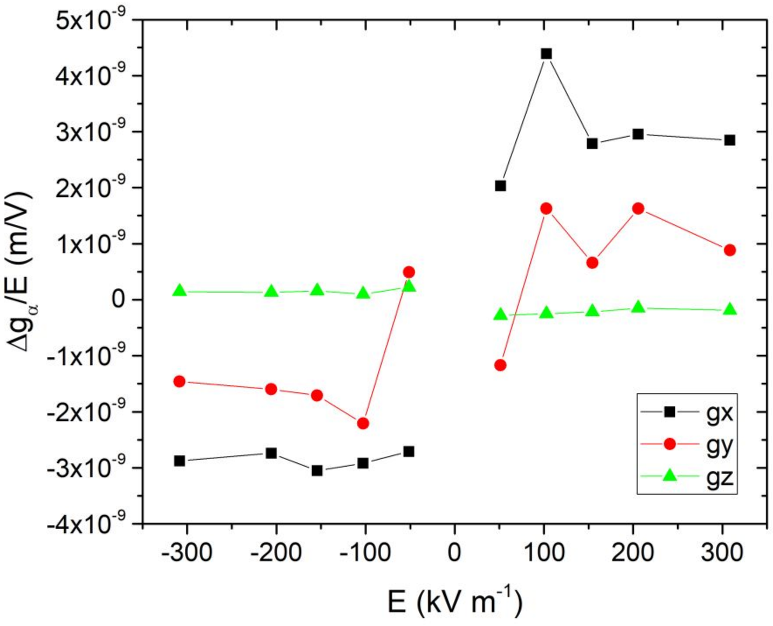

Figure S18 – Variation of the components of the ***g*** factor of the ground doublet associated with the variation of the crystal field parameters shown in Figure S16.

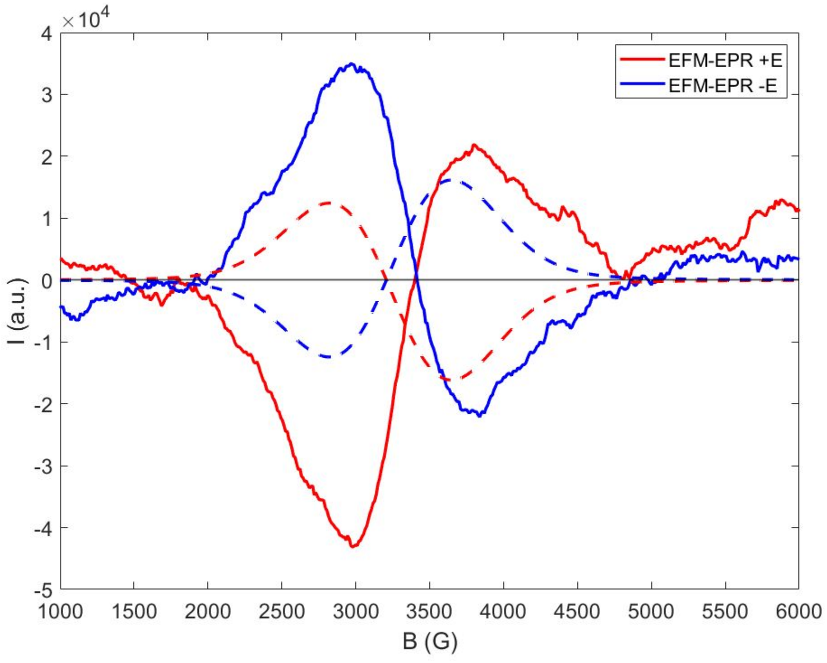

Figure S19 – Experimental EFM-EPR spectra of **Dy(oda)$_3$** with +E and B applied along the crystallographic *b* axis on the Λ enantiomer (continuous lines) and simulated spectra (dashed lines) considering all the ΔB_k^q parameters determined by *ab initio* calculations.

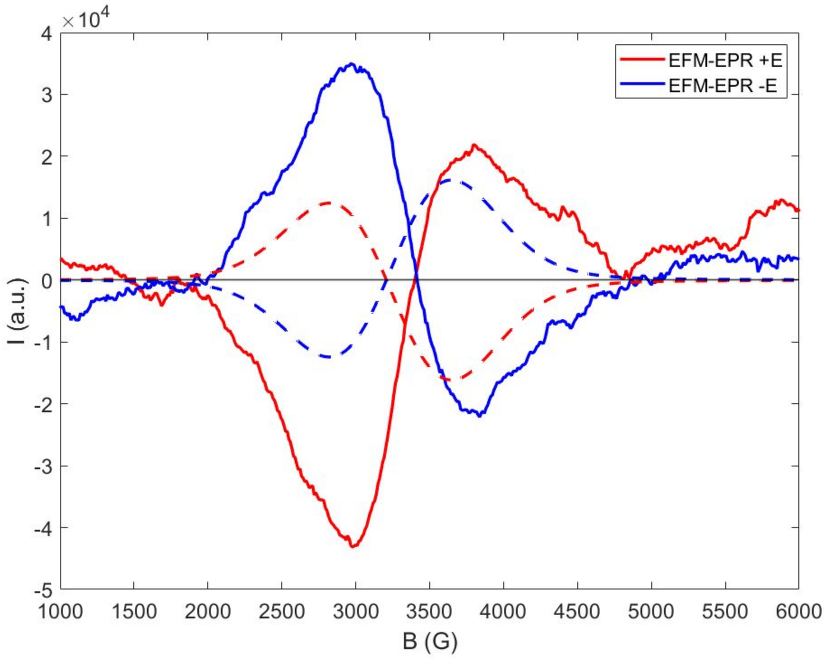

Figure S20 – Experimental EFM-EPR spectra of **Dy(oda)$_3$** with +E and B applied along the crystallographic *b* axis on the Λ enantiomer (continuous lines) and simulated spectra (dashed lines) considering only the ΔB_k^q parameters out of the trigonal symmetry determined by *ab initio* calculations.

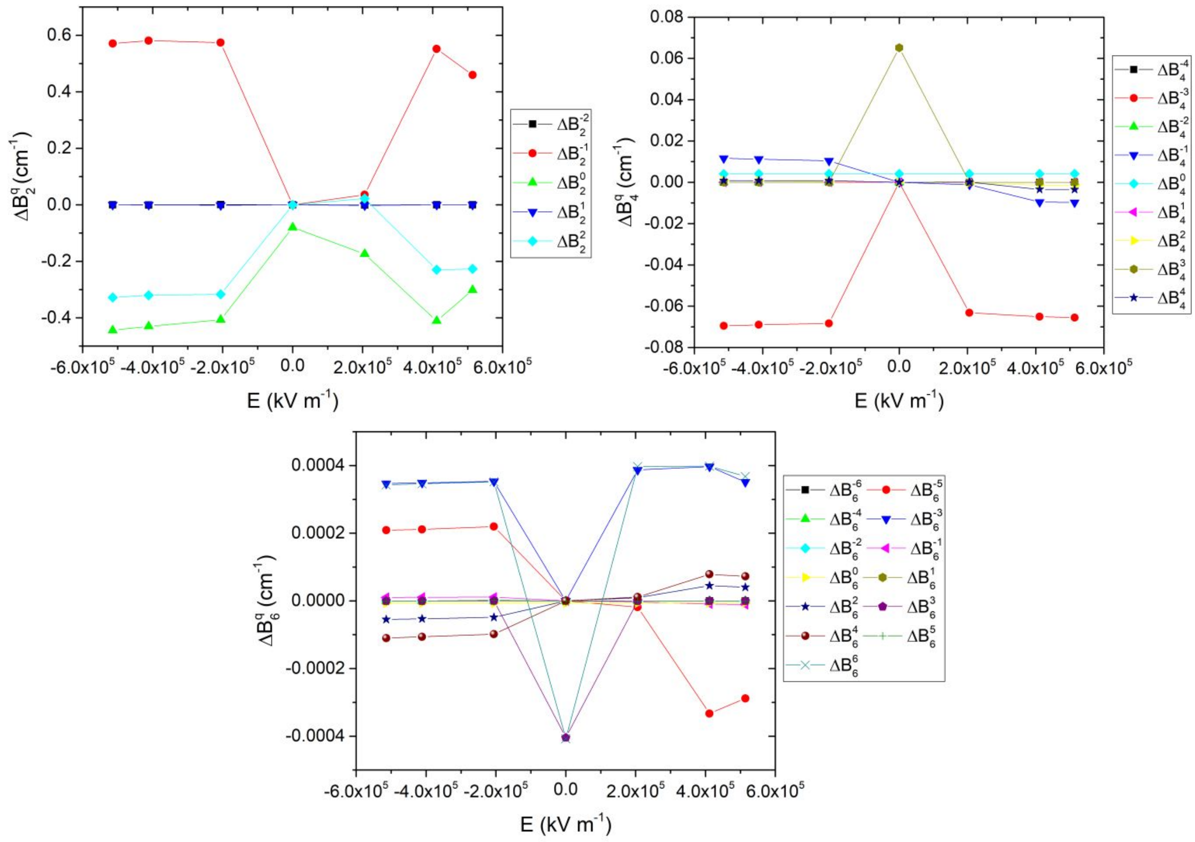

Figure S21 – Variation of the crystal field parameters (ΔB_k^q) as a function of the intensity of applied electric field, determined by *ab initio* calculations on the Λ isomer. Calculations done considering the geometrical perturbation induced by the applied electric field. Positive electric field is applied along the crystallographic -*b* axis.

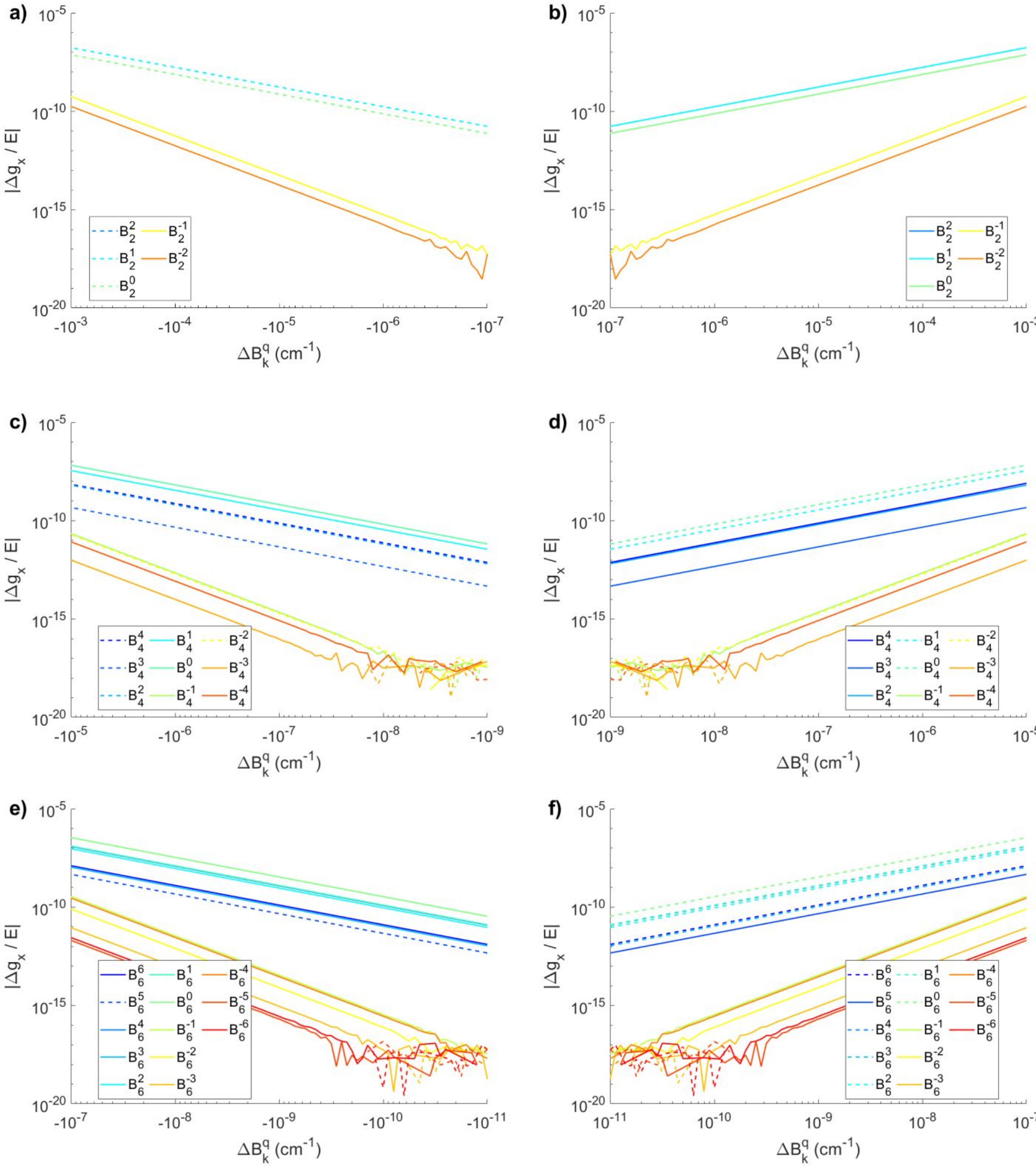

Figure S22 – Modulations of $|\Delta g_x/E|$ calculated by introducing in the unperturbed crystal field Hamiltonian: negative (left column) and positive (right column) variations of crystal field parameters: B_2^q (**a**,**b**); B_4^q (**c**,**d**); B_6^q (**e**,**f**). Solid lines represent positive variations of $\Delta g_x/E$ while dashed lines represent negative variations.

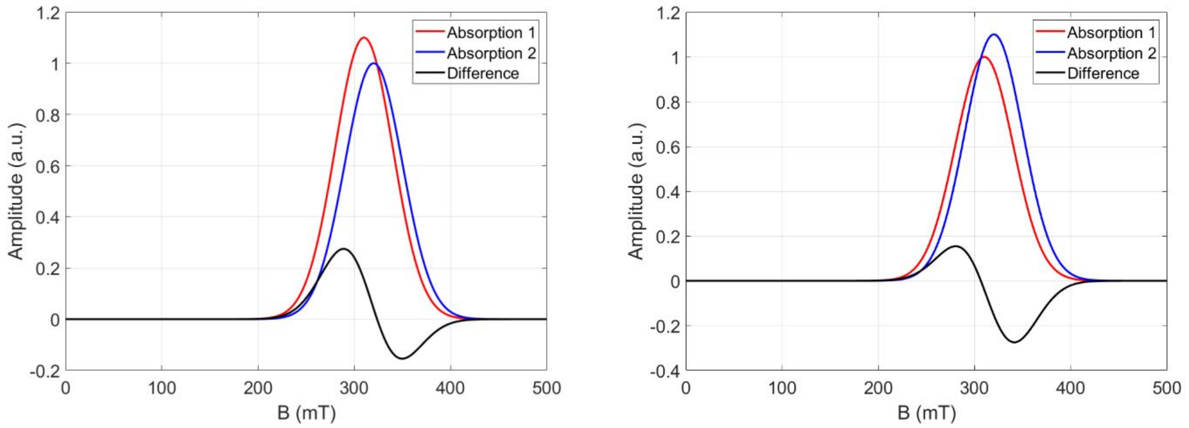

Figure S23 – Resulting asymmetry of the simulated EFM-EPR spectrum considering opposite relative intensities of the two absorption spectra shifted at a higher (red) and lower (blue) g value.

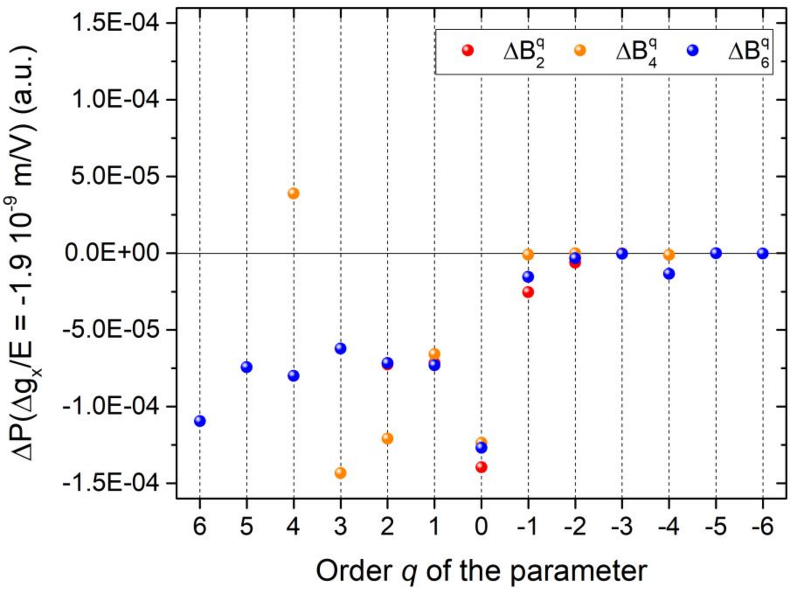

Figure S24 – Variation of the transition probability ΔP associated with variation $\Delta g_x/E = -1.9 \cdot 10^{-9}\ m/V$ for each crystal field parameter, as discussed in main text.

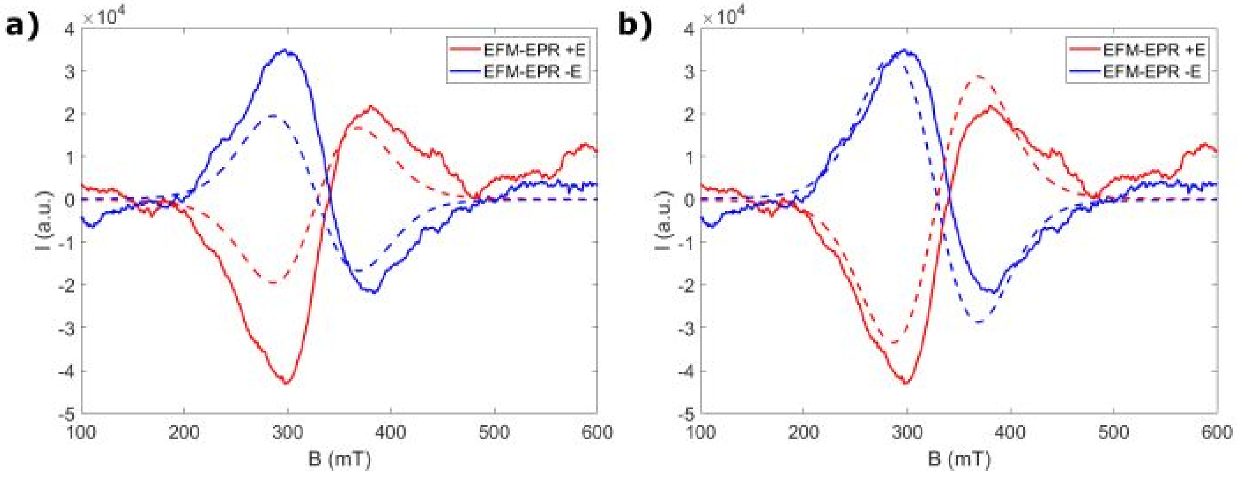

Figure S25 – Experimental (lines) and simulated (dashed lines) EFM-EPR spectra on the Λ enantiomer with the positive electric and magnetic fields applied along the *b* crystallographic axis. Simulations were performed considering a variation of the B_4^4 parameter by the applied electric field equal to $-2.5 \cdot 10^{-6} cm^{-1}$ (**a**) and $-4.3 \cdot 10^{-6} cm^{-1}$ (**b**).

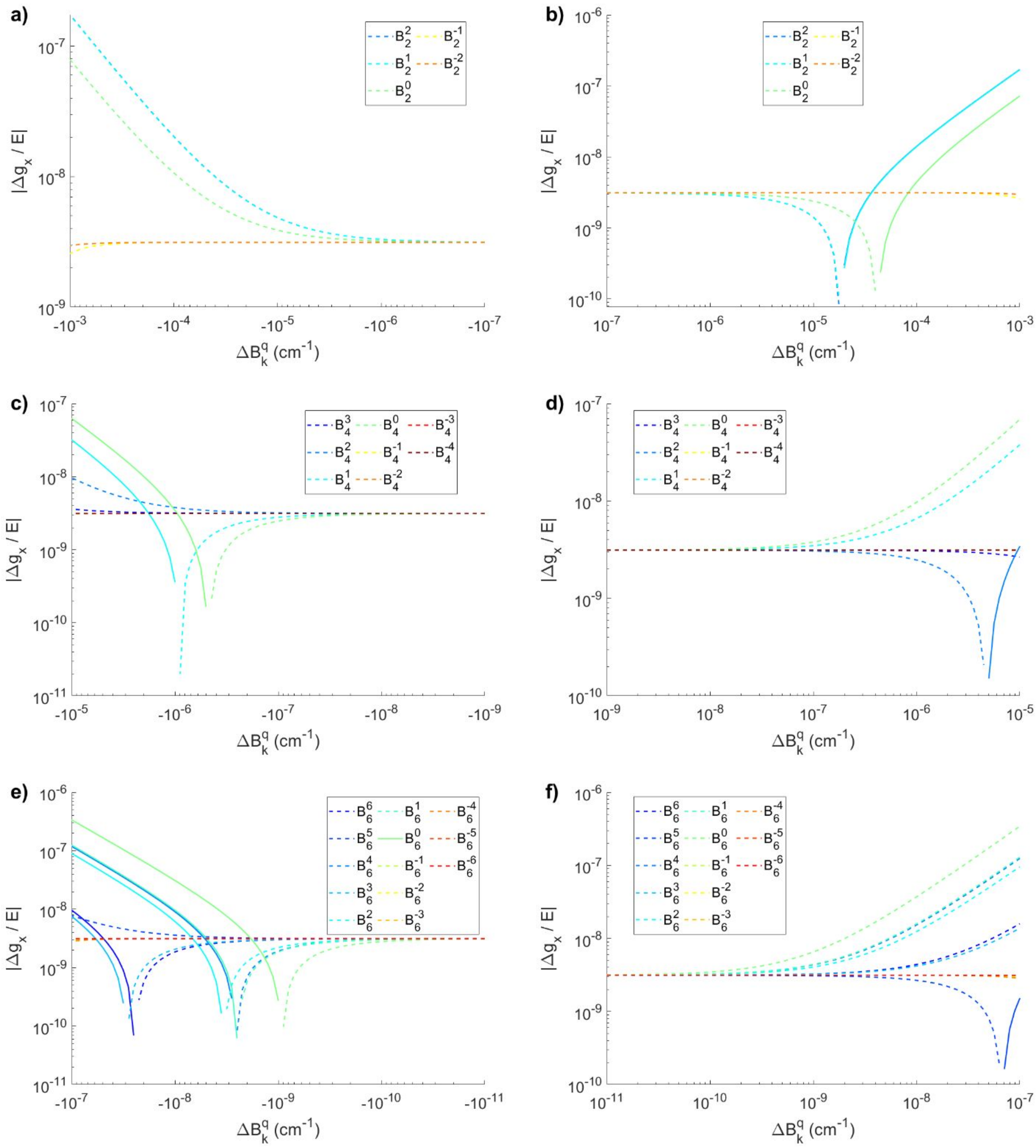

Figure S26 – Modulations of $|\Delta g_x/E|$ by introducing in the unperturbed crystal field Hamiltonian both $\Delta B_4^4 = -4.3 \cdot 10^{-6}$ cm^{-1} and negative (**a**, **c**, **e**) and positive (**b**, **d**, **f**) variations of each crystal field parameters: B_2^q (**a**,**b**); B_4^q (**c**,**d**); B_6^q (**e**,**f**). Solid lines represent positive variations of $\Delta g_x/E$ while dashed lines represent negative variations.

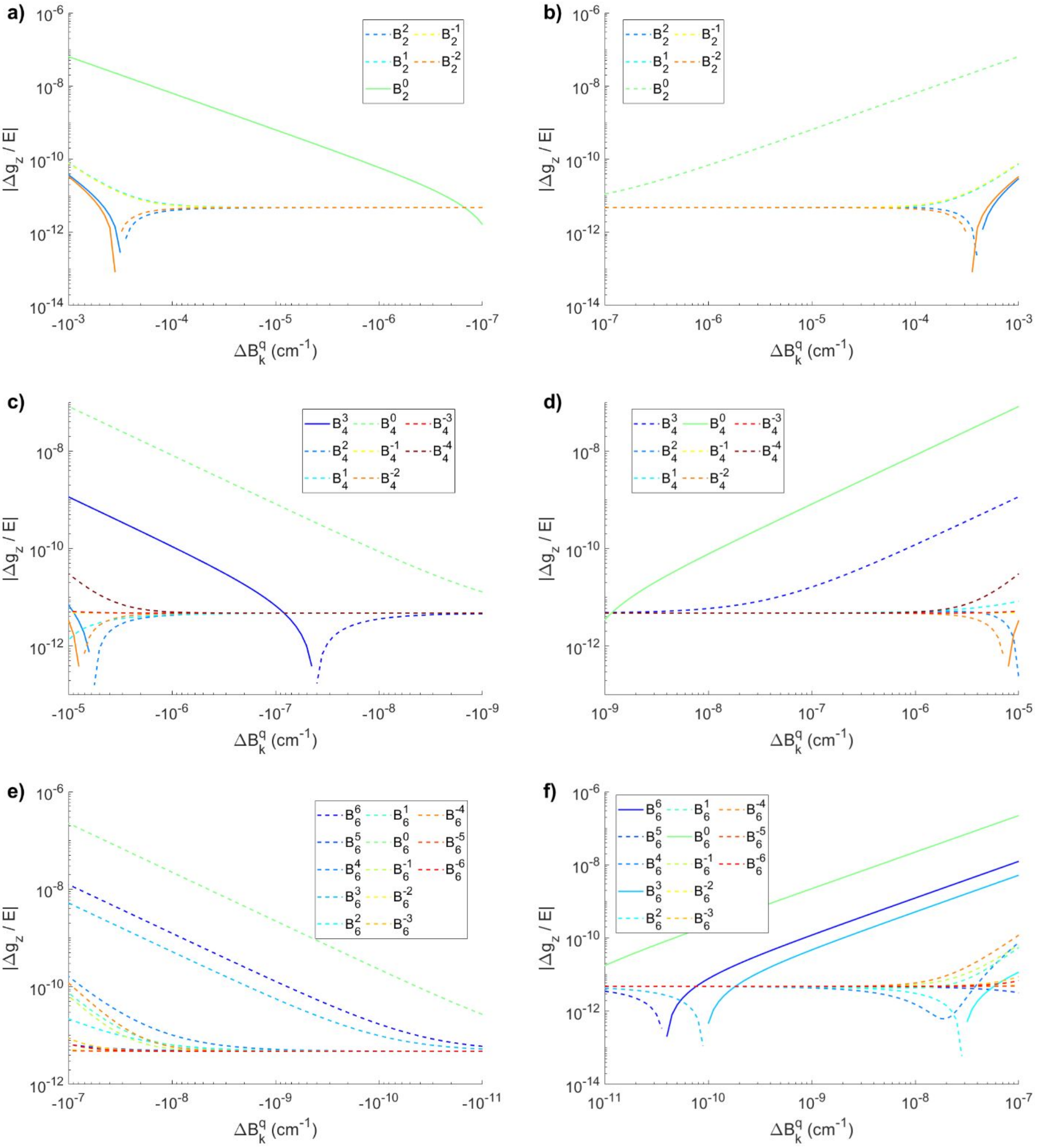

Figure S27 – Modulations of $|\Delta g_z/E|$ by introducing in the unperturbed crystal field Hamiltonian both $\Delta B_4^4 = -4.3 \cdot 10^{-6}$ cm^{-1} and negative (**a**, **c**, **e**) and positive (**b**, **d**, **f**) variations of each crystal field parameters: B_2^q (**a**,**b**); B_4^q (**c**,**d**); B_6^q (**e**,**f**). Solid lines represent positive variations of $\Delta g_z/E$ while dashed lines represent negative variations.

References

(1) Dieke, G. H. *Spectra and Energy Levels of Rare Earth Ions in Crystals*; Crosswhite, H. M., Crosswhite, H., Eds.; Interscience Publishers, 1968.

(2) Metcalf, D. H.; Hopkins, T. A.; Richardson, F. S. Electronic Spectra, Optical Activity, and Crystal-Field Energy-Level Structure of Dy3+ in Trigonal Na3[Dy(Oda)3]*2NaClO4*6H2O Crystals. *Inorg. Chem.* **1995**, *34*, 4868–4878.

(3) Baldoví, J. J.; Duan, Y.; Morales, R.; Gaita-Ariño, A.; Ruiz, E.; Coronado, E. Rational Design of Lanthanoid Single-Ion Magnets: Predictive Power of the Theoretical Models. *Chem. – Eur. J.* **2016**, *22* (38), 13532–13539. https://doi.org/10.1002/chem.201601741.
